# Oncogenic Calreticulin Induces Immune Escape by Stimulating TGF-β Expression and Regulatory T Cell Expansion in the Bone Marrow Microenvironment

**DOI:** 10.1158/0008-5472.CAN-23-3553

**Published:** 2024-09-16

**Authors:** Dominik Schmidt, Cornelia Endres, Rouven Hoefflin, Geoffroy Andrieux, Melissa Zwick, Nikolaos Karantzelis, Hans F. Staehle, Janaki Manoja Vinnakota, Sandra Duquesne, Miriam Mozaffari, Dietmar Pfeifer, Heiko Becker, Bruce R. Blazar, Alexander Zähringer, Justus Duyster, Tilman Brummer, Melanie Boerries, Julian Baumeister, Khalid Shoumariyeh, Juan Li, Anthony R. Green, Florian H. Heidel, Itay Tirosh, Heike L. Pahl, Nils Leimkühler, Natalie Köhler, Marcelo A. S. de Toledo, Steffen Koschmieder, Robert Zeiser

**Affiliations:** 1 Department of Medicine I - Medical centre - University of Freiburg, Faculty of Medicine, University of Freiburg, Germany; 2 Faculty of Biology, Albert-Ludwigs-University, Freiburg, Germany; 3 Department of Molecular Cell Biology, Weizmann Institute of Science, Rehovot, Israel; 4 Institute of Medical Bioinformatics and Systems Medicine, Medical Center - University of Freiburg, Faculty of Medicine, University of Freiburg, Freiburg, Germany; 5 Masonic Cancer Center and Department of Pediatrics, Division of Blood and Marrow Transplantation, University of Minnesota, Minneapolis, Minnesota, USA; 6 IMMZ, University of Freiburg, Faculty of Medicine, University of Freiburg, Germany; 7 German Cancer Consortium (DKTK), Partner site Freiburg, a partnership between DKFZ and Medical Center - University of Freiburg; 8 Department of Hematology, Oncology, Hemostaseology, and Stem Cell Transplantation, Faculty of Medicine, RWTH Aachen University, and Center for Integrated Oncology, Aachen Bonn Cologne Düsseldorf (CIO ABCD), Aachen, Germany; 9 Department of Hematology, University of Cambridge, Cambridge, UK; 10 Department of Hematology, Hemostasis, Oncology and Stem Cell Transplantation, Hannover Medical School (MHH), Hannover, Germany; 11 Leibniz Institute on Aging, Fritz-Lipmann Institute, Jena, Germany; 12 Department of Hematology and Stem Cell Transplantation, University Hospital Essen, Germany; 13 Signalling Research Centres BIOSS and CIBSS – Centre for Integrative Biological Signalling Studies, University of Freiburg

## Abstract

Increasing evidence supports the interplay between oncogenic mutations and immune escape mechanisms. Strategies to counteract the immune escape mediated by oncogenic signaling could provide improved therapeutic options for patients with various malignancies. As mutant calreticulin (CALR) is a common driver of myeloproliferative neoplasms (MPN), we analyzed the impact of oncogenic CALR^del52^ on the bone marrow (BM) microenvironment in MPN. Single-cell RNA-sequencing revealed that CALR^del52^ led to the expansion of Transforming growth factor (TGF)-β1-producing erythroid progenitor cells and promoted the expansion of FoxP3^+^ regulatory T cells (T_reg_) in a murine MPN model. Treatment with an anti-TGF-β antibody improved mouse survival and increased the glycolytic activity in CD4^+^ and CD8^+^ T cells *in vivo*, while T cell depletion abrogated the protective effects conferred by neutralizing TGF-β. TGF-β1 reduced perforin and tumor necrosis factor (TNF)-α production by T cells *in vitro*. TGF-β1 production by CALR^del52^ cells was dependent on JAK1/2, PI3K, and ERK activity, which activated the transcription factor Sp1 to induce TGF-β1 expression. In four independent patient cohorts, TGF-β1 expression was increased in the BM of MPN patients compared to healthy individuals, and the BM of MPN patients contained a higher frequency of T_reg_ compared to healthy individuals. Together, this study identified an ERK/Sp1/TGF-β1 axis in CALR^del52^ MPNs as a mechanism of immunosuppression that can be targeted to elicit T-cell-mediated cytotoxicity.

## Introduction

A connection between oncogenic mutations and immune regulatory mechanisms was shown for different haematological malignancies, such as the c-Myc-PD-L1 axis in lymphoma and multiple others (reviewed in (1)). Philadelphia negative myeloproliferative neoplasms (MPN) typically carry a driver mutation in the genes for janus kinase (JAK) 2, in calreticulin (CALR) or in the thrombopoietin receptor (MPL). All three cause the constitutive activation of downstream JAK / Signal Transducers and Activators of Transcription (STAT) signaling pathway, despite resulting in phenotypically distinct MPN subtypes. *CALR* mutations, primarily associated with essential thrombocythemia (ET) or primary myelofibrosis (MF), occur in about 25% and 35% respectively (2). Current therapeutic options for MPN include hydroxyurea, pegylated interferon alpha or the JAK1/2 inhibitor ruxolitinib which aim to reduce disease related symptoms such as splenomegaly and thromboembolic complications but are rarely curative, as they show little efficacy at antagonizing the natural course of the disease. Allogeneic hematopoietic cell transplantation (allo-HCT) remains the only curative option but comes with the risk for transplant associated mortality and graft-versus-host disease (GVHD) (3). Mutations of the *CALR* gene are located in exon 9. The most prevalent *CALR* mutations are a deletion of 52 base pairs (del52 – type 1) and an insertion of 5 base pairs (ins5 – type 2), both leading to a frameshift altering the C-terminal domain of the protein (2,4) and subsequent MPL-mediated JAK/STAT activation. The impact of these mutations on cells of the immune system residing in the bone marrow (BM) microenvironment is not fully understood. Mechanisms promoting immune escape of MPN and transformation to acute myeloid leukemia include increased PD-L1 expression (5), immune-checkpoint ligand upregulation (6), reduced IL-15 production (7), reduced MHC and TRAIL-receptor expression (8), increased IL-1β production (9) and leukemia-derived lactic acid release (10). Immune escape is a key mechanism for tumor cells to develop drug resistance. Novel strategies to counteract the immune escape which is connected to oncogenic signaling are of high interest to improve our therapeutic options for patients with various malignancies.

TGF-β is involved in therapy resistance and progression of MPN (11). Previous work had shown that TGF-β can induce immunosuppression in the tumor microenvironment of colorectal cancer (12) and that it inhibits phosphorylation and activation of the Tec kinase Itk as well as NFATc translocation in T cells (13). The fusion protein luspatercept, which traps TGF-β ligands thereby preventing them from binding to Type II TGF-β receptors, is already used in clinical routine for myelodysplastic syndrome. A moderate toxicity profile has been reported, indicating that potential strategies to interfere with TGF-β mediated immunosuppression in MPN have reached clinical grade (14). Here, we describe that oncogenic calreticulin activation leads to immune escape in the BM microenvironment via the TGF-β/regulatory T cell (T_reg_) axis. In murine models, TGF-β targeted therapy improved survival but did not eliminate oncogene-carrying cells. Anti-TGF-β therapy improved glycolytic capacity and enhanced TNF-α production in CD4^+^ and CD8^+^ T cells. These data indicate that therapeutic approaches targeting TGF-β or oncogenic signaling leading to the production of TGF-β could enhance elimination of persisting MPN cells.

## Material and Methods

### Human tissue analysis and patients - Study approval

Human peripheral blood or BM samples were collected and their analysis was approved by the Institutional Ethics Review Board of the Medical center, University of Freiburg, Germany (protocol numbers: 10024/13, 558/15). Written informed consent was obtained from each patient. All analyses of human data were carried out in compliance with relevant ethical regulations. The characteristics of patients are listed in Supplementary Table S1.

### Mice - Study approval

The “Institutional Animal Care and Use Committee (IACUC)” has approved our animal studies in the animal protocols **(Protocol numbers: G22-093, G17-061, G-20/096, X-20/07A, X-15/10A)** (Regierungspräsidium Freiburg, regional council), Germany (Federal Ministry for Nature, Environment and Consumers Protection). Mice of the BALB/cAnN (IMSR_APB:4790, H-2K^d^), C57BL/6 (MGI: 2159965, H-2K^b^) or C3H/He (MGI: 6197584 H-2K^k^) strain were obtained at an age of 6-10 weeks from Janvier Labs (France) or from the local stock at the animal facility of Freiburg University Medical Center. Mice were kept at the local animal facility under specific pathogen free (SPF) conditions.

### Viral transduction and primary bone marrow (BM) MPN model

Wild type donor BALB/cAnN mice were treated with 5-FU (100 mg/kg bodyweight – i.p.) four days prior. Bone marrow from tibia, femur and hip bones was isolated and erythrocytes were removed using erythrocyte lysation buffer (NH_4_Cl (0.5M), KHCO_3_ (10mM), EDTA (1mM)). Bone marrow cells were cultured in Dulbecco’s Modified Eagle Medium (DMEM - Gibco) supplemented with 4.5 g/l D-Glucose + L-Glutamin + 10% fetal calf serum (FCS) + 1% Penicillin-Streptomycin + 14.3 ng/ml murine stem cell factor (mSCF – Peprotec - 250-03) + 10 ng/ml murine interleukin-3 (mIL-3 – Peprotec - 213-13) and 10 ng/ml murine interleukin-6 (mIL-6 – Peprotec - 216-16) at 37°C, 5% CO_2_. Viral transduction was performed in three subsequent rounds 12h between each. Bone marrow cells were transferred into 2 ml of fresh virus supernatant media (see virus supernatant harvest) supplemented with 14.3 ng/ml mSCF + 10 ng/ml mIL-3 + 10 ng/ml mIL-6 and 4 µg/ml of Hexadimethrinbromid (Polybrene^®^ - Sigma-Aldrich - TR-1003-G). Cells were centrifuged (2400rpm, 1h 30min, 32°C) and subsequently cultured at 37°C, 5% CO_2_. On day 3 post bone marrow isolation, transduction efficiency was measured using flow cytometry detecting GFP expressing cells. Recipient BALB/cAnN mice were sub-lethally (6.5 Gy split into two doses) irradiated using an IBL637C ^137^Cs source. 100.000-200.000 GFP expressing cells, plus 1*10^6^ wild type BALB/cAnN supportive bone marrow cells were injected intravenously into the tail vein. Engraftment and disease development was monitored by flow cytometry and hemogram analysis (HORIBA Scil Vet ABC) of blood from day 10 onward.

### Mouse model for allogeneic hematopoietic cell transplantation (allo-HCT)

We used a previously described GVL model (7,10). BALB/cAnN recipient mice were irradiated with a total of 10.5 Gy split into two doses of an IBL637C ^137^Cs source. 5 million allogeneic (H2K^b^) bone marrow cells together with 100.000 32D-MPL-CALR^del52^ cells were injected i.v. into the tail vein. On day 2 past bone marrow transplantation additional 200.000 allogeneic T cells (H2K^b^) were injected.

### Anti-TGFβ and cell depletion treatment

After successful engraftment of oncogene bearing hematopoietic cells mice were treated with 10 mg/kg_bodyweight_ anti-murine TGFβ1,2,3 *in vivo* antibody (RRID:AB_2921436) or mouse IgG1 isotype control (RRID:AB_2921382) on day 12,16 and 20 post BM transplantation intraperitoneal for TGFβ neutralizing therapy. For adaptive immune cell depletion mice were treated with 250 µg of anti-mouse CD4 *in vivo* (RRID:AB_2921444) and anti-mouse CD8 in vivo (RRID:AB_2921445) depletion antibody or Rat IgG2b isotype control (RRID:AB_2921378) starting from day 10 post bone marrow transplantation, day 14 and subsequently once per week until end of the experiment.

### JAK2^V617F^ knock-in and Calr^del52^ knock-in mice

JAK2^V617F^ knock-in mouse strain (16) was generous gifted by Jean-Luc Villeval and crossed with *MX1cre* transgenic mice (Tg(Mx1-cre)1Cgn: IMSR_JAX:003556) in SPF conditions at the animal facility of Freiburg University Medical center. Bone marrow of 7-13 week old Mx1cre-Jak2^V617F^ mice or JAK2^WT^ littermates was analyzed by flow cytometry.

CALR^del52^ knock-in mouse strain was previously described (17). Mice were kept in SPF conditions at the animal facility of the Department of Haematology, University of Cambridge, Cambridge, United Kingdom. Bone marrow of 10-12 months old mice was analyzed at the local site.

### Cell lines

The murine myeloid cell line 32D (RRID:CVCL_0118) and the murine pro B cell line Ba/F3 (RRID:CVCL_0161) were purchased from DSMZ and cultured in Roswell Park Memorial Institute (RPMI) 1640 Medium + 10% fetal calf serum (FCS) + 1% Penicillin-Streptomycin (P/S) + 5 ng/ml murine interleukin-3 (mIL-3). Cells were kept at a density between 0.2-1.5*10^6^ cells/ml. 32D cells and Ba/F3 cells were authenticated by highly polymorphic short tandem repeat analysis (performed by Microsynth) latest tested in 04/2024 and 05/2023, respectively. There was no detectable contamination with human origin. The retroviral packaging cell line Plat-E (RRID:CVCL_B488) and the T lymphoblast cell line EL-4 (RRID:CVCL_0255) were purchased from Cell Biolabs and ATCC, respectively. Cells were cultured in DMEM (Gibco) supplemented with 4.5 g/l D-Glucose + L-Glutamin + 10% FCS + 1% P/S. Plat-E cells were cultured for two weeks in DMEM medium (10%FCS, 1% P/S) supplemented with 1µg/ml Puromycin (InvivoGen - ant-pr-1) and 10µg/ml Blasticidin S - hydrochlorid (Sigma Aldrich – 15205) for selection of packaging vector expressing cells. All cells were tested negative for mycoplasma and cultured for at least one week before any experiments were performed. All cells were cultured at 37°C, 5% C0_2_.

### Starvation and inhibitor treatment of cell lines

For IL-3 starvation and inhibitor treatment experiments 32D cells and Ba/F3 cells were harvested and washed with PBS two consecutive times to remove any remaining IL-3. Cells were seeded at a density of 400.000 cells/ml in a 6-Well plate, 2.5 ml final volume. Inhibitors or DMSO were added for the indicated concentrations. 32D cells were treated with Buparlisib (BKM120 – selleckchem), Pictilisib (GDC-0941 - MedChem Express), Selumetinib (AZD6244 - MedChem Express), Trametinib (GSK1120212 - selleckchem), Ulixertinib (BVD-523 - selleckchem), STAT3 Inhibitor VI (S3I-201, Calbiochem), Plicamycin (MedChem Express), AP-1 inhibitor (T-5224 - MedChem Express), Elk-1 inhibitor (TAT-DEF-Elk - MedChem Express), CREB inhibitor (666-15 - MedChem Express), FOXO1 inhibitor (AS1842856 - MedChem Express) or NFκB inhibitor (JSH-23 - MedChem Express) as indicated. Cells were kept under experimental conditions overnight, 37°C, 5% C0_2._

### Culture of primary cells

T cells were isolated from spleens of healthy donor mice by magnetic labeling and subsequent cell separation using Pan T cell isolation kit II (Miltenyi Biotec, 130-095-130) according to manufacturer instructions. Cells were cultured in RPMI 1640 Medium + 10% FCS + 1% P/S + 2-Mercaptoethanol (25µM). T cells were activated using mouse-T-Activator CD3/CD28 Dynabeads™ (Gibco™ - 11456D). T cells were cultured at an initial density of 1*10^6^ cells/ml.

### Isolation of human peripheral blood mononuclear cells (PBMCs) and bone marrow cells

Written informed consent was obtained from each healthy donor or patient. PBMCs were collected from peripheral veins and collected in EDTA monovettes^®^ (SARSTEDT). Bone marrow was collected by bone marrow puncture and supplemented with EDTA. Blood or bone marrow was diluted with phosphate buffered saline (PBS) 1:3 or 1:2 respectively and layered onto lymphocyte separation media (anprotec, AC-AF-0018). Density gradient centrifugation was performed (30min, RT, 440g with brakes turned off). Mononuclear layer was collected and cells were washed two times with PBS. Cells were counted. 5*10^6^ cells were frozen in fetal calf serum (FCS) + 10 % DMSO, 10*10^6^ cells were taken for RNA isolation and/or protein isolation, 0.5*10^6^ cells were taken for flow cytometry analysis.

### MPL and TGFB expression on iPSC-derived erythroid cells

MPN patient-specific induced pluripotent stem cells (iPSC) harboring the CALR del52 mutation in heterozygosis and the respective CRISPR-repaired CALR WT iPSC lines have been previously described (18). iPSC lines were cultivated on Matrigel-coated (Corning) 6-well plates and differentiated towards the hematopoietic lineage using the spin-EB method (18,19). For the differentiation towards the erythroid lineage, hematopoietic progenitor cells at day 14 of the Spin-EB differentiation were seeded (15.000 cells/ml, 1 ml per 35 mm dish) in Methylcellulose (StemCell Technologies) supplemented with 20 ml IMDM (Gibco) and 50 ng/ml rhSCF, 10 ng/ml rhIL-3, 10 ng/ml rhGM-CSF and 14 ng/ml rhEPO (all Immunotools). After 10-12 days at 37°C, 5% C0_2_ and 95% humidity, CFUs were harvested, washed 2X with PBS 1X (Gibco) and subjected to flow cytometry analysis.

### Immunocytochemistry and image cytometry

Cells were fixed using 4% formaldehyde, permeabilized and blocked using 5% BSA and goat serum + 0.1% TritonX100 and incubated with Anti-phospho STAT3 Tyr705 (Cell Signaling, RRID:AB_331586) for 1h. Goat-anti-rabbit IgG (H+L) AlexaFluorPlus647 secondary antibody (Invitrogen, RRID:AB_2633282) was added for 1h. Nuclei were stained using DAPI and high-content screening/image cytometry was performed using Olympus ScanR microscope (UPLSAPO 20x): Continuous ZDC was used and 36 images per well were taken automatically. Using Olympus ScanR analysis 3.4.1 software, cells were analyzed. New assay was set up and nucleus was defined as main object. Watershed was enabled and thresholds were set individually per experiment. Sub-objects were defined based on main object. Mean intensities were calculated using total intensity/area.

### Luciferase promotor assay

2*10^6^ 32D-MPL-Calr^WT/del52^ cells were seeded into 6-Well plate prior to transduction. 2 µg of pGL3-TGFb1 vector and 0.2 µg of pGL4.73(hRluc/SV40) Vector (Promega - E6911) were transfected into pre-seeded cells using Lipofectamin™2000 transfection reagent (Invitrogen™) according to manufacture instructions. Cells were cultured for 24h in transfection mix supplemented with IL-3. IL-3 and transfection mix was removed from culture and 0.4*10^6^ cells were seeded in a 12-Well plate in fresh culture medium supplemented with inhibitors or DMSO control. STAT3-Inhibitor VI (S3I-201 - Calbiochem), ERK inhibitor (Ulixertinib BVD-523 - selleckchem) and SP-1 inhibitor (Plicamycin - MedChem Express) were used at indicated concentrations. Cells were cultured overnight. Luciferase and hRluc activity was measured using Dual-Glo® Luciferase Assay System (Promega - E2920) using Spark 10M (Tecan) Luminescence Multi Mode Microplate Reader for detection of luminescence signal. PGL3-TGFb1 was a gift from Yuh-Shan Jou (Addgene plasmid # 101762 ; http://n2t.net/addgene:101762 ; RRID:Addgene_101762) (20).

### Flow cytometry and fluorescence activated cell sorting

All antibodies and reagents used are listed in Supplementary Table S2. If not otherwise stated flow cytometry stains were performed as following. For cytokine staining’s cells were treated with protein transport inhibitor – Golgi-Plug™ containing Brefeldin A (BD Bioscience) for 4h at 37°C. Dead cells were excluded using LIVE/DEAD Fixable Dead Cell Stain kit (Molecular Probes, USA) or LIVE/DEAD™ Fixable Aqua Dead Cell Stain Kit (Thermo Scientific), 20 min 1:500. To avoid unspecific antibody binding, Fc receptor was blocked using human FcR Blocking Solution (Miltenyi Biotec, RRID:AB_2892112 – 1:5 10 min) or murine True Stain FcX (BioLegend, RRID:AB_2818986 – 1:25, 10 min). Extracellular antibody mix was added directly to cells in the Fc receptor blocking solution for 30 min. For intracellular or nuclear staining the cells were fixed using Fixation/Permeabilization Kit (BD Bioscience - RRID:AB_2869008) or Foxp3/Transcription Factor Staining Buffer Set (eBioscience™ - 00-5523-00) according to manufacturer instructions. Intracellular antibody staining was performed in buffers provided with the respective kit, O/N or 1h at 4°C.

### Single-cell energetic metabolism by profiling translation inhibition (SCENITH)

To analyze metabolic profiles of different T cell subsets we used a method published by Argüello et al called SCENITH (21). Primary bone marrow (BM) MPN or transplantation control mice were sacrificed and splenocytes isolated. Erythrocytes were depleted using erythrocyte lysis buffer and cells were counted. 5*10^6^ splenocytes for each condition were plated in a 96 well plate in 50 µl of RPMI (10%FCS, 1% P/S) and set to rest from 1h at 37°C. 50 µl of Inhibitor cocktail was added directly onto the splenocytes at a final concentration of 100 mM 2-Deoxy-D-Glucose (Sigma - D8375), 1 µM Oligomycin (Sigma - 1404-19-9) – incubate 15 minutes at 37°C. 100 µl of pre-diluted O-Propargyl-Puromycin (OPP) reagent was added directly to the cells (final dilution factor 1:2000) and incubated for 30 minutes at 37°C. Flow cytometry staining was performed like described above and cells were fixed using Fixation/Permeabilization Kit (BD Bioscience - RRID:AB_2869008). Click reaction was performed using Click-iT™ Plus OPP Alexa Fluor™ 647 Protein Synthesis Assay Kit (Invitrogen™ - C10458) following manufacture instructions. Fluorescence activated cell sorting and detection of OPP incorporation was performed using BD LSR Fortessa. Geometric mean of Alexa Fluor™ 647 signal in target population was calculated as a value for OPP incorporation. Glycolytic capacity, mitochondrial dependency, fatty acid oxidation / amino acid oxidation (FAO/AAO) capacity and glucose dependency were calculated as previously described (21).

### RNA isolation, cDNA synthesis and quantitative PCR

RNA from single cell suspension was isolated using the RNeasy^®^ Mini Kit (Qiagen - 74104) according to manufactures instructions. On column DNase digestion was performed to reduce DNA contamination. RNA concentration and quality was measured using NanoDrop1000 spectrophotometer (PeqLab, RRID:SCR_016517). cDNA was synthesized using High Capacity cDNA Reverse Transcription Kit (Applied Biosystems) with a total of 1000 ng of RNA in a final volume of 20 µl. Cycler was programmed to: STEP 1 – 25°C, 10min; STEP 2 – 37°C, 120min; STEP 3 – 85°C, 5min. cDNA was diluted with RNase free water to a final concentration of 25ng/µl. Quantitative PCR was performed using LightCycler 480 SYBR Green I Master Kit (Roche) and the respective qPCR primer pair (Supplementary Table S3). 1 µl of cDNA template was used and a separate dilution series was prepared for each analyzed gene. Quantitative-PCR reaction was performed using Roche LightCycler 480 Real Time PCR System (RRID:SCR_018626). Cp values and PCR efficiency were calculated using LightCycler® 480 SW 1.5.1 Software (Roche).

### Single cell RNA sequencing

Primary BM MPN mice or empty vector control mice where sacrificed on day 21 past bone marrow injection and bone marrow cells were isolated. Erythrocytes were removed and cells were counted. Samples were processed using the Chromium Next GEM Single Cell 3’ Reagent Kit v3.1 (Dual Index – 10x Genomics) following manufactures protocol. In short: GEM´s were generated using a Chromium Controller (10x Genomics, RRID:SCR_019326) at a target cell recovery rate of 10.000. GEM-RT and subsequent clean-up was performed. cDNA was amplified and 3’ Gene Expression Dual Index Library Construction was performed. Illumina paired-end, dual indexing was performed. For all amplification steps a Veriti 96 Well Thermal Cycler (appledbiosystems - RRID:SCR_021097) and for all quality control steps a 2200 TapeStation (Agilent Technologies - RRID:SCR_014994) was used.

### Single cell RNA-seq analysis

For each sample, we excluded cells that had fewer than 600 detected genes, considering them as low-quality. Next, we converted the Unique Molecular Identifier (UMI) matrix into Counts Per Million (CPM) by normalizing each gene’s count based on the total number of UMIs per sample. We applied log2 transformation with the formula log2(CPM/10+1). Next, we centered the data by subtracting the mean expression value of each gene from all its corresponding values. We applied a further filter, retaining only genes with an average expression greater than 4 in log2(CPM) across all cells. The resulting gene-cell matrix underwent dimension reduction using top 100 principal components of a principal component analysis (PCA) and was fed into the Uniform Manifold Approximation and Projection (UMAP) algorithm for visualization. Clustering was performed using nearest-neighbor graph construction (k=20) and graph-based clustering using the louvain method. We calculated differentially expressed genes for every cluster and used the top 50 genes as signatures to assess enrichments with published signatures for mouse bone marrow (22–24) by hypergeometric test (FDR adjusted p<0.01 was considered significant).

The code for the scRNA sequencing analysis is available on the GitHub repository (RRID:SCR_002630, DOI:https://github.com/RouvenHoefflin/Schmidt_et_al_2024/tree/main).

### Bulk RNA analysis

RNA of 2*10^6^ overnight IL-3 starved 32D-MPL-CALR^WT^/CALR^ins5^ cells was isolated using miRNeasy® Mini Kit (Qiagen) according to manufactures instructions. RNA quality and integrity was confirmed using a Fragment Analyzer (Advanced Analytical Technologies, Inc. Ames, IA). Applied Biosystems GeneChipTM WT PLUS Reagent Kit (catalog n.: 902280) was used according to manufactures instructions for library preparation. Probes were hybridized to Affymetrix Clariom S arrays (Affymetrix, USA). The arrays were normalized via robust multichip averaging as implemented in the R/Bioconductor oligo package.

### Protein analysis by western blot

For protein pathway analysis 32D cells were cultured overnight in IL3 starvation conditions at a density of 0.2*10^6^ cells/ml. 2*10^6^ cells were lysed in radioimmunoprecipitation assay (RIPA) buffer (Santa Cruz Biotechnology) supplemented with Phosphatase Inhibitor Cocktail 2 (1861281 - Sigma-Aldrich). Cells were lysed for 20 minutes on ice and cell fragments removed by centrifugation (10 minutes, 10.000g, 4°C). Protein concentration was measured using BCA protein assay (Thermo Scientific™) according to manufacture protocol. 5-15 µg protein was prepared with sample reducing agent (Invitrogen™) and LDS-sample buffer (Invitrogen™) and heated at 70°C, 10 minutes. SDS-PAGE was performed using NuPAGE Bis-Tris 4-12% gradient gel (Invitrogen™) and NuPAGE MES/MOPS SDS running buffer (homemade). Separated protein were transferred onto a PVDF Transfer Membranes, 0.45 µm (Thermo Scientific™) – 20V, 70 minutes. Primary antibody staining was performed overnight at 4°C and respective secondary antibody, linked with HRP enzyme, stained for 1h at RT. HRP signal was detected using WesternBright Quantum HRP substrate (Advansta) and imaging was performed using ChemoStar M6 (Intas Science Imaging Instruments GmbH) camera.

### Retroviral system and generation of retroviral supernatant

We used a previously reported CALR MUT-transformed -MPL cells (15). For all transductions we used the Murine Stem Cell Virus (MSCV) retroviral expression vector system. We used MSCV backbone plasmid with different oncogenes and IRES-GFP (pMIG - Addgene_9044) or hygromycin resistance gene, cloned into multiple cloning site (Supplementary Table S4). For generation of virus supernatant we used Platinum-E (Plat-E, RRID:CVCL_B488) retroviral packaging cell line. MSCV vectors were transfected using Lipofectamine™2000 (Invitrogen™ - 11668019) transfection reagent according to manufacture protocol. Transfection was checked using fluorescence microscopy after 24h and transfection mix was replaced by 5 ml of fresh Dulbecco’s Modified Eagle Medium (DMEM - Gibco) supplemented with 4.5 g/l D-Glucose + L-Glutamin + 10% FCS + 1% P/S. Virus supernatant was collected three subsequent times with 12h between. Virus supernatant was sterile filtered using a 0.45µm syringe filter and stored for less than one week at 4°C.

### Quantification of TGFB1 expression in publicly available single-cell RNA-sequencing dataset (GSE156644)

We used the publicly available single-cell RNA-sequencing datasets comparing bone marrow stromal cells in myelofibrosis and healthy bone-marrow (25). In short, the datasets were generated from FACS-purified cells isolated from native bone marrow biopsies, which were subsequently subjected to droplet-based sc-RNA-sequencing (Chromium Single Cell 3′, v2 chemistry) (25,26). The published R-object was downloaded via ZENODO (RRID:SCR_004129, https://doi.org/10.5281/zenodo.3979087) and data integrity verified (27). Marker genes were determined using the FindMarkers function as supplied by the Seurat R-package (28). Genes with an adjusted p-value below 0.05 and a positive normalized gene expression were considered as significantly differentially expressed. Probability density of transcript-specific expression per cell cluster was plotted by calling the RidgePlot function after subsetting the dataset based on the assigned cluster identity.

### Statistical analysis

For the sample size in the murine experiments a power analysis was performed. A sample size of at least n=8 per group was determined by 80% power to reach a statistical significance of 0.05 to detect an effect size of at least 1.06. Differences in animal survival (Kaplan-Meier survival curves) were analyzed by Mantel Cox test. The experiments were performed in a non-blinded fashion. All samples or mice were included in our analysis.

All data were tested for normality applying the Kolmogorov-Smirnov test. For statistical analysis of 2 groups an unpaired two tailed Student’s *t test* was applied. If the data did not meet the criteria of normality, the Mann-Whitney *U* test was applied. If more than 2 groups were analyzed we used the Kruskal-Wallis-Test if non-parametric testing was suggested and we performed a one-way ANOVA in case of normally distributed data. For grouped data sets with several variables, we used two-way ANOVA. Statistical analysis was performed using GraphPad Prism (RRID:SCR_002798). Data are presented as mean and s.e.m. (error bars). Differences were considered significant when the *P*-value was <0.05.

Raw microarray data (.CEL) files were processed with the oligo R package (RRID:SCR_015729, (29)). Samples were normalized using the Robust Multichip Average preprocessing. Differential gene expression analysis was performed with the linear model-based approach (limma R package, RRID:SCR_010943, (30)) comparing samples from different condition with unpaired design matrix. Ajusted *P*-value (Benjamini Hochberg) below 0.05 was considered as significant. Microarray data downloaded from the Gene Expression Omnibus (GEO) were processed in the exact same way. Signalling pathway activity was quantified using decoupleR R package (31) with the Progeny annotation (32) as input network. The weighted arithmetic mean (wmean) was computed for each pathways from the t-statistics of the differential gene expression analysis as input (i.e mat) together with the following parameters: times = 100, minsize = 5.

### Data Availability Statement

Microarray data that we generated are available in the GEO at GSE241162. Single cell RNA sequencing data are deposited in the database GSE241364. All data are deposited in the super series GSE241365. The code for the scRNA sequencing analysis and the scRNA data are available on the GitHub repository (RRID:SCR_002630, DOI:https://github.com/RouvenHoefflin/Schmidt_et_al_2024/tree/main) and on ZENODO (RRID:SCR_004129, DOI:https://zenodo.org/doi/10.5281/zenodo.11311826), respectively.

We have analyzed publicly available datasets. The publicly available data analyzed in this study were obtained from Gene Expression Omnibus (GEO) at GSE156644, GSE174060 and GSE214361.

All other raw data are available upon request from the corresponding author.

## Results

### Mutant CALR induces TGF-β production

**Figure 1:**
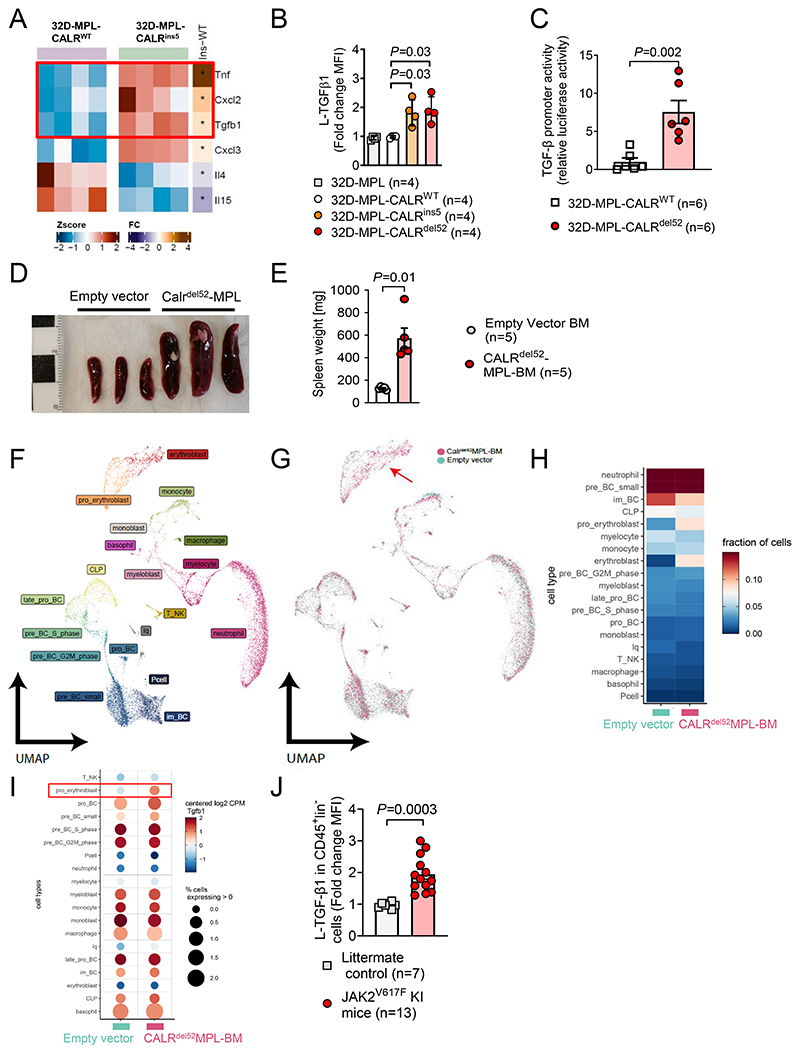
Mutant CALR induces TGF-β production **(A)** Heat map showing expression profile of MPL-CALR^ins5^ transduced 32D cells versus MPL-CALR^WT^. Relative expression of selective immune regulatory cytokines is plotted. Colour codes represents the Z-score log2 intensity. RNA was harvested from four independent IL3-starved cell cultures. Differential gene expression analysis was performed with the linear model-based approach (limma R package). **(B)** Scatter plot showing fold change of MFI from L-TGF-β1 expressed on MPL-CALR^-/WT/del52^ transduced 32D cells after IL-3 withdrawal. Four independent experiments were performed and the results were pooled. *P*-values were calculated using one-way ANOVA. **(C)** Scatter plot showing TGF-β promoter activity (luciferase activity relative to mean of WT control) of 32D-MPL-Calr^WT^ or 32D-MPL-Calr^del52^ cells. Cells transfected with the pGL3-TGFβ1 promotor vector. Pooled data from six independent experiments. *P* values were calculated using Mann-Whitney test. **(D, E)** Spleens of mice isolated 21 days after injection of either Empty vector (EV) or CALR^del52^-MPL transduced Bone Marrow (BM). Exemplary picture (scale in cm) (D) and scatter plot (E) showing quantification of weight of spleens of mice 21 days after injection of either EV- or CALR^del52^-MPL transduced BM. **(F-I)** Single-cell RNA Sequencing (sc-RNA-Seq) of bone marrow from mice 21 days after injection of either EV- (n=2) or CALR^del52^-MPL (n=2) transduced BM. UMAP depicting clustering into different cell populations (**F**), UMAP of EV- and CALR^del52^-MPL condition merged (**G**), heat-map depicting fraction of cells in each cluster (**H**) and bubble plot depicting TGF-β1 expression in different clusters combining fraction cells expressing TGF-β1 (% - relative expression to mean >0) and expression within clusters relative to mean expression level over all clusters (**I**). The red arrow indicates the erythroblast population in bone marrow of mice that received CALR^del52^-MPL BMC. **(J)** Scatter plot showing TGF-β protein expression (fold change of MFI) of CD45^+^ lineage marker negative cells isolated from JAK2^V617F^ knock-in mice or littermate controls as indicated. Each data point is a biological replicate (individual mouse). *P* values were calculated using an unpaired Student’s t-test (E, J).

To study the impact of mutant calreticulin (CALR), we used non-malignant 32D cells expressing human thrombopoietin receptor (MPL) and induced either human *CALR*^WT^ (control), *CALR*^ins5^ or *CALR*^del52^ oncogenes (15). Microarray-based transcriptome analysis revealed more than 6000 differentially regulated genes (Supplementary Figure S1A). Among these, we found upregulation of TGF-β1 RNA together with pro-inflammatory cytokines including TNF-α and Cxcl2 (Figure 1A). Interestingly, we found several downstream signaling transducers of TGF-β to be downregulated including Smad1, 4 and 7, while TGF-β signaling inhibitor Smurf1 was upregulated in oncogene-transduced 32D cells, suggesting that an inhibitory feedback mechanism was induced, thereby preventing autocrine TGF-β signaling (Supplementary Figure S1B). Increased latency-associated-peptide-TGF-β1 (L-TGF-β1) protein expression was detected in MPL-CALR^ins5^ and MPL-CALR^del52^ mutant 32D cells compared to MPL-CALR^WT^ cells or empty vector control cells (Figure 1B). MPL-CALR^ins5^ and MPL-CALR^del52^ induced L-TGF-β1 production was confirmed in the IL3-dependent pro-B cell line Ba/F3 (Supplementary Figure S1C). Additionally, increased TGF-β protein production was observed in 32D cells expressing MPL and *JAK2*^*V617F*^ (Supplementary Figure S1D). This indicates that myeloid cells with active JAK2/STAT signaling produce TGF-β. When exposed to JAK1/2 inhibition TGF-β protein was reduced in BA/F3 cells expressing MPL and *JAK2*^*V617F*^ (Supplementary Figure S1E). To identify the transcriptional mechanism of how TGF-β is upregulated we used a TGF-β promoter reporter assay and observed that reporter activity for the TGF-β gene was increased in 32D-MPL-Calr^del52^ cells compared to 32D-MPL-CALR^WT^ cells (Figure 1C). This indicates that MPL-CALR^del52^ induces increased transcription of TGF-β.

Bone morphogenetic protein (BMP) pathway receptors and their ligands are closely associated with TGF-β family members. Therefore, we studied if MPL-CALR^del52^ was connected to changes in BMP receptors and ligands. We observed that BMP members Bmpr2 and Acvr1b were downregulated in 32D-MPL-Calr^del52^cells compared to 32D-MPL-CALR^WT^ cells (Supplementary Figure S2).

To characterize the impact of CALR^del52^ on the immunological microenvironment, we transfected primary BM cells with human *MPL* and human *CALR*^*del52*^ and transferred the BM into mice that had undergone total body irradiation. Disease progression was monitored by peripheral blood counts (Supplementary Figure S3A-D). When mice developed splenomegaly and increased hematocrit (Figure 1D-E, Supplementary Figure S3D), BM isolated from two mice from each group was analyzed by single-cell RNA sequencing (scRNA-seq). We first clustered all cells and assigned them to cell types using differentially expressed marker genes (Figure 1F, Supplementary Figure S3E).

Cluster distribution was similar between empty vector and CALR^del52^MPL-BM group with the exception of a higher fraction of cells in the erythroblast and pro-erythroblast clusters within the CALR^del52^MPL-BM group (Figure 1F-H). Next, we compared TGF-β expression in clusters of both groups (Figure 1I). We observed an increased absolute count of TGF-β-expressing erythroid progenitor cells within the CALR^del52^MPL-BM group compared to the empty vector control group (Figure 1I). We used transduced primary mouse BM cells expressing *MPL* and *CALR*^*del52*^ which caused the differential gene expression. Our approach corresponds to the physiological situation where MPL is expressed in erythroid progenitor cells and its activation contributes to erythropoiesis (33).

We could not find any genes related to immunosuppression that were significantly differently regulated in cells carrying CALR^del52^ other than TGF-β.

JAK2^V617F^ and CALR^del52^ both induce similar oncogenic JAK2/STAT1/3 signaling. To understand if only CALR^del52^ or also JAK2^V617F^ can induce TGF-β production, we used JAK2^V617F^ knock-in (KI) mice that were previously reported (5,16). We observed that TGF-β protein increased in CD45^+^ cells in the BM of JAK2^V617F^ KI mice compared to littermate control mice (Figure 1J). This indicates that not only erythroid progenitor cells but also CD45^+^ linage^-^ cells produce TGF-β protein upon oncogenic JAK2/STAT1/3 activation. Additionally, CALR^del52^ and JAK2^V617F^ both induce TGF-β expression.

**Figure 2:**
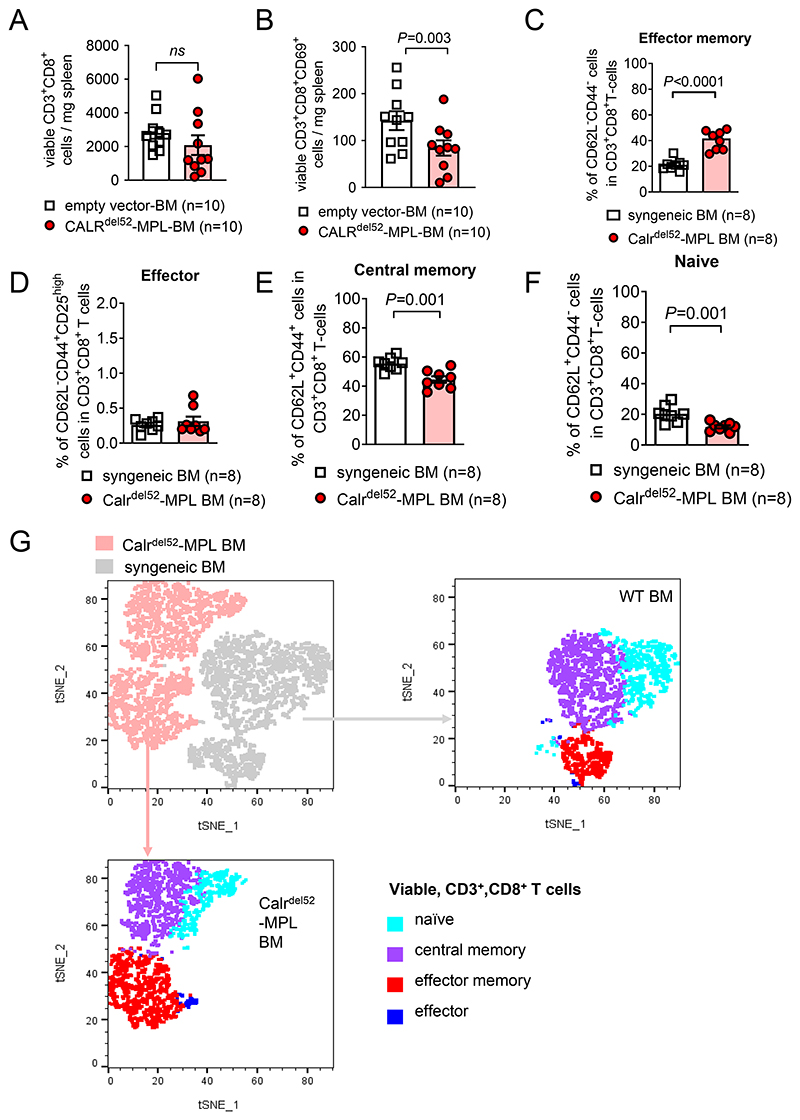
T cell phenotype in mice with CALR^del52^-MPL or control BM **(A, B)** Scatter plot showing absolute counts of total CD3^+^CD8^+^ T cells (A) and CD3^+^CD8^+^CD69^+^ activated T cells (B) isolated from the spleens of mice injected with BM cells expressing CALR^del52^-MPL or control BM. Pooled data from two individual experiments. *P* values were calculated using unpaired Student’s t-test. **(C-F)** Scatter plot showing frequencies of effector memory (CD62L^-^ CD44^-^) T cells (C), effector (CD62L^-^ CD44^+^) T cells (D), central memory (CD62L^+^ CD44^+^) T cells (E) and naïve (CD62L^+^ CD44^-^) T cells (F) in total CD3^+^CD8^+^ viable T cells isolated from the spleens of mice injected with BM cells expressing CALR^del52^-MPL or control BM. Pooled data from two individual experiments. *P* values were calculated using unpaired Student’s t-test. **(G)** Representative dot plot showing t-distributed stochastic neighbour embedding (t-SNE) of viable CD3^+^CD8^+^ T cells derived from the spleens of mice injected with BM cells expressing CALR^del52^-MPL or control BM. For visualization, previously gated T cell subsets (C-F) are superimposed as indicated.

Since TGF-β was shown to be essential for the differentiation of tissue-resident memory CD8^+^ T cells but also suppresses effector T cell function (34), we next analyzed CD8^+^ T cells in the spleens of mice that had received BM containing empty vector or CALR^del52^MPL-BM. Total CD8^+^ T cell count did not change in mice that had received BM containing CALR^del52^MPL-BM compared to empty vector, however, we found a significant decrease in the absolute count of CD8^+^ cytotoxic T cells expressing the activation marker CD69 (Figure 2A, B). Conversely, the frequency of effector memory T cells was increased in the CALR^del52^MPL-BM group compared to the syngeneic BM control group (Figure 2C). Central memory T cells and naïve T cells were decreased in the CALR^del52^MPL-BM group compared to the syngeneic BM control group (Figure 2D-G).

### *In vivo* TGF-β1,2,3 neutralization improves survival of CALR^del52^ driven MPN

**Figure 3:**
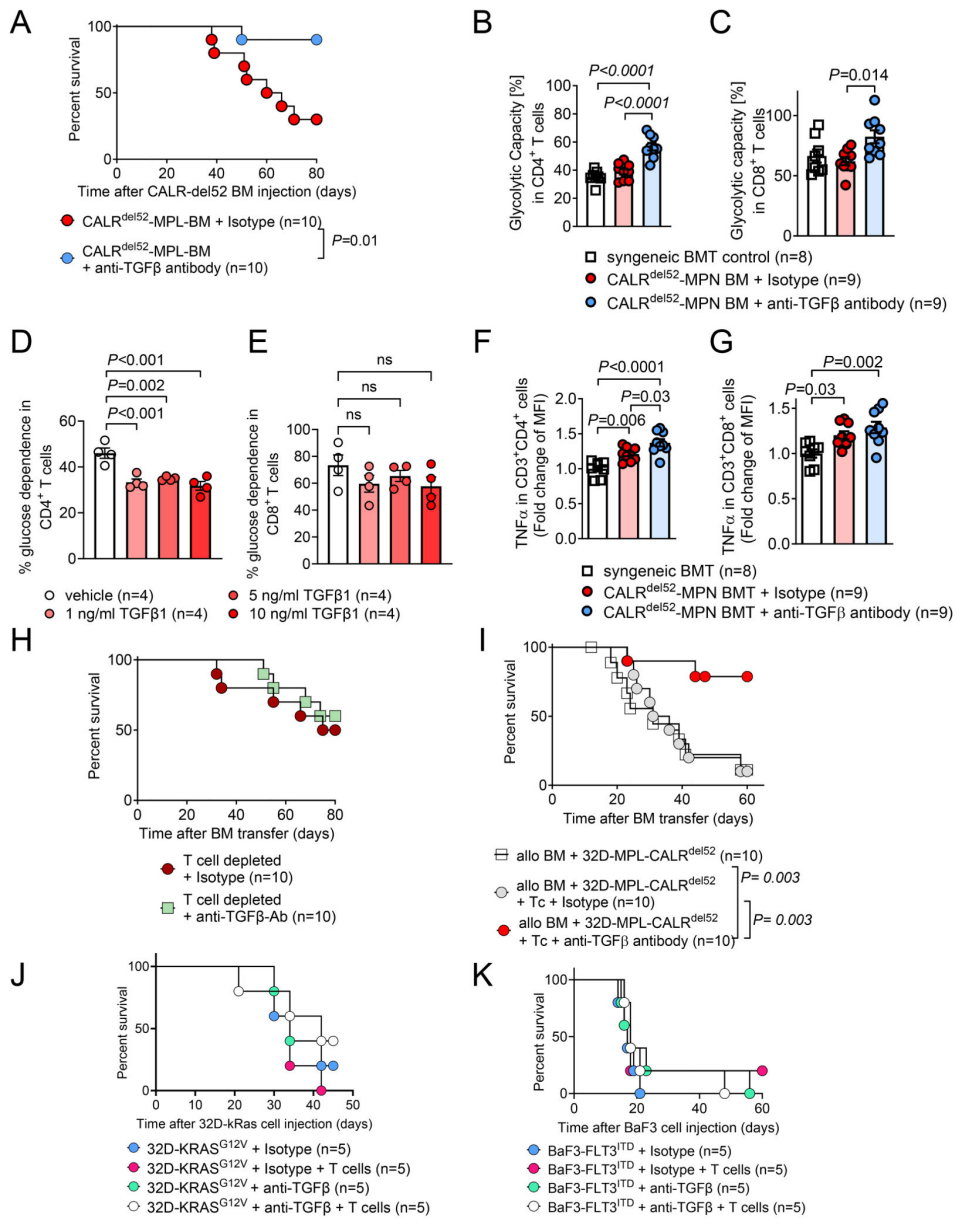
*In vivo* TGF-β neutralization improves survival of CALR^del52^ driven MPN **(A)** Kaplan-Meyer curve showing percent survival of mice injected with BM cells expressing CALR^del52^-MPL. Mice were treated with anti-TGFβ1,2,3 neutralizing antibody or respective isotype controls on day 12, 16 and 20 post bone marrow transplantation. Pooled data from two individual experiments. **(B, C)** Scatter plot showing glycolytic capacity of CD4^+^ (B) and CD8^+^ (C) T cells isolated from spleen of mice injected with syngeneic BM or syngeneic BM cells expressing CALR^del52^-MPL. Mice were treated with anti-TGFβ1,2,3 neutralizing antibody or respective isotype controls on day 12, 16 and 20 post bone marrow transplantation. Pooled data from two individual experiments. **(D, E)** Scatter plot showing glucose dependence (%) in CD4^+^ (D) and CD8^+^ (E) T cells activated using CD3/CD28 activator beads and treated with indicated concentrations of TGF-β for 24h. Pooled data from four individual experiments. **(F, G)** Scatter plot showing fold change of TNF-α MFI in CD3^+^CD4^+^ (F) and CD3^+^CD8^+^ (G) T cells isolated from spleen of mice injected with syngeneic BM or syngeneic BM cells expressing CALR^del52^-MPL. Mice were treated with anti-TGFβ1,2,3 neutralizing antibody or respective isotype control on day 12, 16 and 20 post bone marrow transplantation. Pooled data from two individual experiments. **(H)** Kaplan-Meyer curve showing percent survival of mice injected with BM cells expressing CALR^del52^-MPL. Mice received weekly T cell depleting therapy (anti-CD4 and anti-CD8 antibody) and were treated with anti-TGFβ1,2,3 neutralizing antibody or respective isotype control on day 12, 16 and 20 post bone marrow transplantation. Pooled data from two individual experiments. **(I)** Kaplan-Meyer curve showing percent survival of mice injected with allogeneic BM and 32D-MPL-CALR^del52^ cells. Mice received 2x10^5^ allogeneic T cells (Tc) on day 2 post allo-HCT and were treated with anti-TGFβ1,2,3 neutralizing antibody or respective isotype control on day 12, 16 and 20 post allo-HCT, as indicated. Pooled data from two individual experiments. **(J)** Kaplan-Meyer curve showing percent survival of mice injected with 32D-KRAS^G12V^ cells and allogeneic BM. Mice received 2x10^5^ allogeneic T cells (Tc) on day 2 post allo-HCT and were treated with anti-TGFβ1,2,3 neutralizing antibody or respective isotype control on day 12, 16 and 20 post allo-HCT, as indicated. Data from one experiment is shown. **(K)** Kaplan-Meyer curve showing percent survival of mice injected with BaF3-FLT3^ITD^ cells and allogeneic BM. Mice received 200.000 allogeneic T cells (Tc) on day 2 post allo-HCT and were treated with anti-TGFβ1,2,3 neutralizing antibody or respective isotype control on day 12, 16 and 20 post allo-HCT, as indicated. Data from one experiment is shown. *P* values were calculated using Mantel-Cox Test (A, H-K) or ordinary one-way ANOVA (B-G). Bar plots show mean at SEM.

To clarify the functional relevance of TGF-β *in vivo*, we treated mice carrying CALR^del52^MPL BM with an anti-TGFβ1,2,3 (henceforth referred as anti-TGFβ) neutralizing antibody. We observed an improved survival of mice upon TGF-β neutralization using anti-TGF-β antibody treatment on days 12, 16 and 20 (intraperitoneal) after transplantation (Figure 3A). Using single-cell metabolomics based analysis with the SCENITH method (21), we observed an increase in glycolytic capacity in CD4^+^ and CD8^+^ T cells isolated from the spleens of mice that had received CALR^del52^MPL-BM and TGF-β neutralization compared to isotype treated control (Figure 3B, C). Higher glycolytic capacity suggests more efficient effector T cell function including interferon-γ production and tumor cell lysis (10).

To determine the glycolytic activity of T cells in the presence or absence of TGF-β, we used a controlled *in vitro* system. We observed that TGF-β induced a reduction of the glucose dependency in CD4^+^ T cells *in vitro*, while in CD8^+^ T cells we observed only a trend towards a reduction of glucose dependency upon TGF-β exposure (Figure 3D, E).

We observed upregulation of TNF-α in CD4^+^ and CD8^+^ T cells of mice that had received CALR^del52^MPL-BM compared to syngeneic control mice (Figure 3F, G), which supports the hypothesis that TNF-α production in T cells is induced by the CALR^del52^MPL-driven BM cells.

This finding is consistent with prior work showing that TNF-α is increased in JAK2^V617F^ MPN (35), which we now also found in CALR^del52^MPL-driven MPN. It was shown that TNF-α levels correlated with the JAK2^V617F^ allele burden and exposure of JAK2^V617F^-positive cells to a JAK inhibitor caused reduced TNF-α transcription (35). Using TNF-α deficient mice, the authors could demonstrate that TNF-α was required for the development of the MPN-like disease (35). These results and our findings support that TNF-α has tumor-promoting effects in MPN.

When mice were treated with neutralizing anti-TGF-β antibody, we observed an additional increase of TNF-α expression in CD4^+^ helper T cells compared to mice treated with isotype control (Figure 3F). To validate this finding we used a TGF-β-receptor specific small molecule inhibitor. We observed that TGF-βR inhibition increased TNF-α expression in the mouse EL-4 lymphocyte cell line and primary mouse T cells in a dose dependent manner (Supplementary Figure S4A, B).

To examine T cell function in MPN progression, we depleted CD4^+^ and CD8^+^ T cell populations, using CD4/CD8 specific antibodies, (Supplementary Figure S 5A, B) in mice that had received CALR^del52^MPL-BM and treated these with a TGF-β neutralizing antibody. T cell depletion abrogated the beneficial effect on survival induced by TGF-β neutralization in mice that had received CALR^del52^MPL-BM (Figure 3H). MPN to AML transition (secondary AML, sAML) is a key step in disease progression and is driven by the accumulation of oncogenic mutations. The only curative therapy option for most patients with sAML remains allo-HCT. Patients with sAML experience a worse overall survival, leukemia-free survival and GVHD/relapse-free survival compared to *de novo* AML patients after allo-HCT (36). To test TGF-β neutralization in an allo-HCT setting, we transplanted oncogene (MPL-CALR^del52^) driven 32D cells together with allogeneic BM and consecutive allogeneic T cells on day two. Mice that received allo-HCT together with 32D-MPL-CALR^del52^ cells and TGF-β-neutralizing therapy showed a better overall survival compared isotype control or allo-HCT / 32D-MPL-Calr^del52^ only control (Figure 3I).

To understand if the protective effect of TGF-β-neutralizing therapy was specific for the CALR^del52^ mutation we used additional models that rely on 32D cells transduced with KRAS^G12V^ or FLT3^ITD^ transgenic BaF3 cells and studied the therapeutic efficacy of TGF-β inhibition *in vivo*. We observed that TGF-β-neutralizing therapy did not improve survival of mice carrying 32D cells transduced with KRAS^G12V^ or FLT3^ITD^ transgenic BaF3 cells (Figure 3J, K).

These findings identify TGF-β neutralization as an option for Calr^del52^-driven MPN and as a potential combination treatment after allo-HCT for sAML.

### TGF-β induces regulatory T cells *in vitro* and is connected to a higher frequency of T_reg_ cells *in vivo*

**Figure 4:**
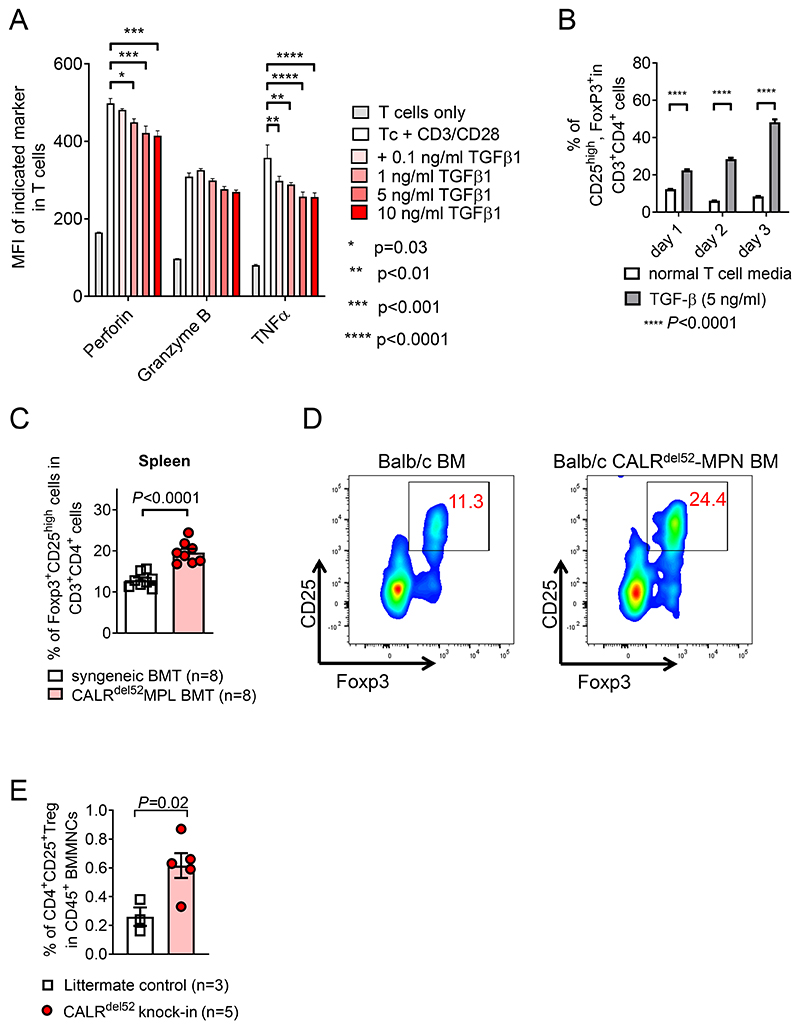
TGF-β induces regulatory T cells while reducing cytotoxic molecules in T cells **(A)** Bar plot showing mean fluorescence intensity (MFI) of Perforin, Granzyme B and TNF-α in T cells after 24h of incubation with CD3/CD28 activation beads and indicated concentration of soluble TGFβ1. Experiment was repeated three times, technical triplicates of one exemplary repeat is depicted. *P* values were calculated using 2way ANOVA with multiple comparison. **(B)** Bar plot showing percentage of CD25^high^Foxp3^+^ T_regs_ in CD3^+^CD4^+^ T cells treated with 5 ng/ml of soluble TGF-β or normal T cell media and activated with CD3/CD28 activation beads, for the indicated time. Experiment was repeated three times, technical triplicates of one exemplary repeat is depicted. *P* values were calculated using 2way ANOVA with multiple comparison. **(C, D)** Scatter plot (**C**) and exemplary flow cytometry plot (**D**) showing percentage of CD25^high^Foxp3^+^ T_regs_ in CD3^+^CD4^+^ T cells in the spleen of mice injected with BM cells expressing CALR^del52^-MPL or control BM, day 21 post BM transplantation. Pooled data from two individual experiments. *P* values were calculated using unpaired Student’s t-test. **(E)** Scatter plot showing fraction of CD4^+^CD25^+^ T_reg_ cells within total CD45^+^ BM mononuclear cells (BMMNCs) derived from mice homozygous for CALR^del52^ knock-in allele or litter mate control mice. *P* values were calculated using unpaired Student’s *t test*. Bar plots show mean with SEM.

To validate our finding that TGF-β plays a central role in MPN mediated T cell inhibition in a controlled system, we analyzed differentiation of naïve T cells *in vitro*. We exposed splenic T cells isolated from C57B/6 cells to increasing concentrations of TGF-β in vitro for 24h. The T cells were either stimulated with CD3/CD28 or left unstimulated for 24h. Afterwards, T cell differentiation was analyzed by flow-cytometry. The T cell activation system had been reported previously (37). *In vitro* TGF-β exposure reduced expression of perforin and TNF-α in T cells, while there was only a trend for reduced granzyme B expression in T cells upon exposure to TGF-β during CD3/CD28 based activation (Figure 4A). TGF-β led to an increased frequency of CD25^+^Foxp3^+^ T_reg_ within CD4 T cells over time when T cells were cultivated *in vitro* (Figure 4B). Consistent with the findings made *in vitro*, we detected an increased frequency of T_reg_ cells in spleens of mice that received CALR^del52^MPL-BM compared to control BM on day 21 post BM transplantation (Figure 4C, D). We analyzed the frequency of T_reg_ cells in CALR^del52^-knock-in mice that were previously described (17) to validate our findings. CALR^del52^-knock-in mice exhibited an increase in CD4^+^CD25^high^ T_reg_ cells in the bone marrow compared to littermate control mice (Figure 4E). These results support the immunosuppressive effect of TGF-β on T cells, a mechanism that could promote immune evasion.

### CALR^del52^ induces TGF-β via JAK2 and ERK signaling

**Figure 5:**
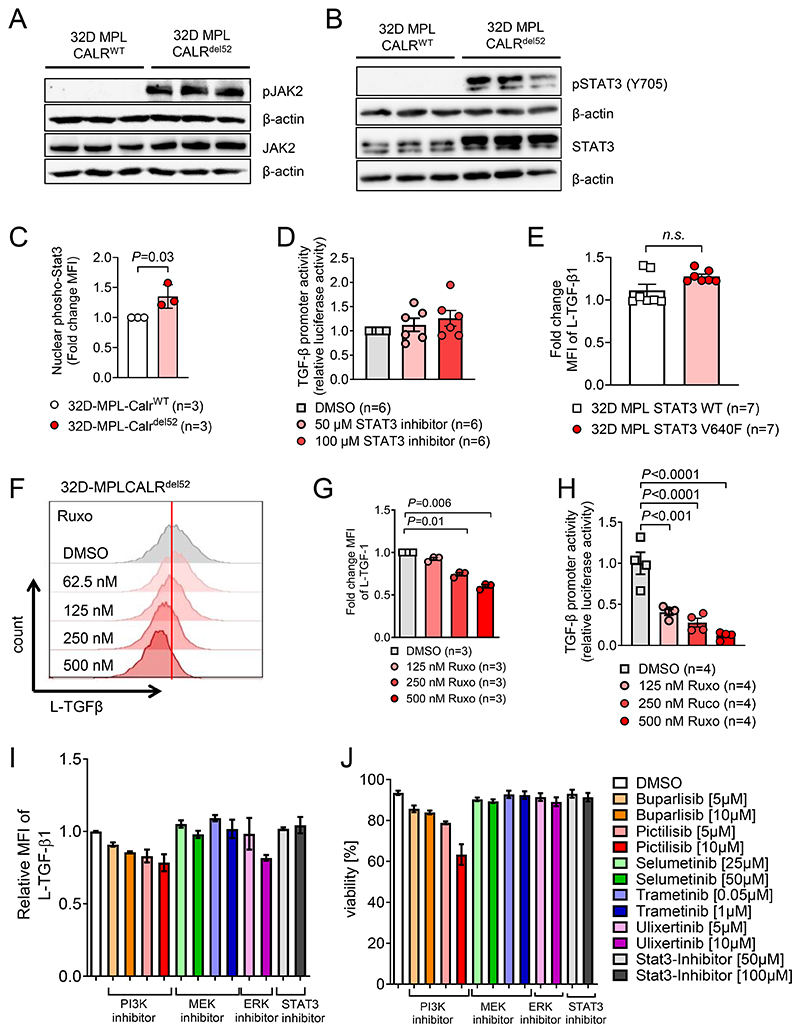
TGF-β expression is regulated by a PI3K/ERK dependent pathway **(A, B)** Western blot showing phospho-JAK2 / total JAK2 (A) and phospho-STAT3 (Y705) /total STAT3 (B) with β-actin as loading control. Protein was isolated from 32D cells transfected with MPL and CALR^WT^ or CALR^del52^ after overnight IL3 starvation, three individual harvest for each condition. **(C)** Scatter dot plot showing fold change of nuclear phospho-STAT3 signal detected by immunohistochemistry in 32D-MPL-CALR^del52^ cells compared to 32D-MPL-CALR^WT^ cells after overnight IL3 starvation. Pooled data from three independent experiments. **(D)** Scatter plot showing luciferase activity in 32D-MPL-Calr^del52^ cells, transfected with pGL3-TGFβ1 promotor vector, treated overnight with indicated concentrations of STAT3 inhibitor after IL-3 withdrawal. Pooled data from two independent experiments. *P* values were calculated using repeated-measures (RM) one-way ANOVA. **(E)** Scatter plot showing fold change MFI of L-TGF-β1 on 32D-STAT3^WT^ or STAT3^V640F^. Pooled data from two independent experiments. *P* values were calculated using Mann-Whitney test. **(F, G)** Representative histogram (**F**) and scatter dot plot (**G**) showing L-TGF-β1 expression on 32D-MPL-CALR^del52^ cells treated with indicated concentration of ruxolitinib (JAK1/2-inhibitor). Pooled data from three independent experiments. **(H)** Scatter dot plot showing relative luciferase activity in 32D-MPL-Calr^del52^, transfected with pGL3-TGFβ1 promotor vector and treated with indicated concentration of ruxolitinib overnight after IL-3 withdrawal. Pooled data from two independent experiments with two technical replicates for each condition. *P* values were calculated using ordinary one-way ANOVA. **(I, J)** Relative expression of L-TGF-β1 (**I**) and cell viability (**J**) of 32D-MPL-CALR^del52^ cells treated with either PI3K inhibitors (Buparlisib, Pictilisib), MEK inhibitors (Selumetinib, Trametinib), ERK inhibitor (Ulixertinib) or STAT3-inhibitor for 24h. Pooled data from three independent experiments.

To dissect how mutant CALR induces TGF-β expression, we performed signaling pathway analysis. We first assessed if MPL-CALR^del52^ is able to induce JAK2/STAT3 signaling. Parental 32D cells require IL-3 to grow and proliferate, but become independent of IL-3 when transduced with MPL and oncogenic *CALR* (15). We confirmed these findings and show IL-3-independent JAK2/STAT3 signaling (Figure 5A, B) and proliferation (Supplementary Figure S6A) of 32D cells expressing mutant CALR^del52^ but not of CALR^WT^ cells. The STAT3 moiety Y705 was highly phosphorylated in 32D-MPL-CALR^del52^ cells. The same pattern of JAK2/STAT3 activation was seen when 32D-MPL-CALR^ins5^ were compared to 32D-MPL-CALR^WT^ cells (Supplementary Figure S6B).

To test the role of STAT3 activation for TGF-β transcription we next analyzed nuclear localization of pSTAT3 using immunofluorescence microscopy. We observed that pSTAT3 nuclear localization increased in 32D-MPL-CALR^del52^ cells compared to CALR^WT^ cells (Figure 5C, Supplementary Figure S6C). STAT3 inhibition did not reduce TGF-β promoter activity in 32D-MPL-Calr^del52^ (Figure 5D). Consistent with the concept that STAT3 activation was not involved in TGF-β transcription, we observed that TGF-β protein production was not increased in 32D cells carrying a STAT3 gain-of-function (V640F) mutation (Figure 5E).

To test which signaling pathways induce TGF-β expression, we treated 32D-MPL-CALR^del52^ cells with inhibitors of JAK1/2 signaling pathways. TGF-β1 expression decreased in 32D-MPL-CALR^del52^ cells treated with the JAK1/2 inhibitor ruxolitinib (Figure 5F, G). We used a TGF-β promoter reporter assay and observed that reporter activity for the TGF-β gene was decreased in 32D-MPL-Calr^del52^ cells when exposed to ruxolitinib in a concentration-dependent manner (Figure 5H). This indicates that JAK1/2 signaling are required for transcription of TGF-β in 32D-MPL-Calr^del52^ cells.

Next, we used specific inhibitors for downstream targets of JAK1 and JAK2 and analyzed L-TGF-β1 expression. When 32D-MPL-CALR^del52^ cells were treated with the PI3K inhibitors buparlisib or pictilisib, we found reduced L-TGF-β1 expression (Figure 5I) alongside reduced viability (Figure 5J). It had been shown that mutant JAK2 activity can induce PI3K and further downstream MAPK dependent pathways (38). We found reduced L-TGF-β1 expression when 32D-MPL-CALR^del52^ cells were treated with the ERK inhibitor ulixertinib (Figure 5I). In contrast treatment with a STAT3 inhibitor or a MEK inhibitor did not reduce L-TGF-β1 expression (Figure 5I). PI3K-signaling leads to activation of mammalian target of rapamycin (mTOR) known as the PI3K/AKT/mTOR pathway (39). We found increased levels of total mTOR and phospho mTOR in 32D-MPL-CALR^del52^ cells compared to 32D-MPL-CALR^WT^ cells (Supplementary Figure S7A-B), which motivated us to test whether mTOR signaling is required for CALR^del52^-induced expression of L-TGF-β1. Treatment of 32D-MPL-CALR^del52^ with the mTOR inhibitor rapamycin did not decrease the expression of L-TGF-β1 (Supplementary Figure S7C), indicating that mTOR signaling was dispensable for the connection between CALR^del52^ and TGF-β. We subsequently treated 32D-MPL-CALR^del52^ cells with an anti-TGF-β antibody and studied JAK downstream target activity. Treatment of 32D-MPL-CALR^del52^ cells with anti-TGF-β antibody reduced the expression of L-TGF-β1 in a dose-dependent manner (Supplementary Figure S7D-E). *In vitro* anti-TGF-β treatment caused reduced STAT3 and ERK activation (Supplementary Figure S8A-D) in 32D-MPL-CALR^del52^ cells indicating an inhibitory effect of anti-TGF-β treatment on CALR^del52^ induced signaling pathways.

Both findings support the hypothesis that autocrine TGF-β1 induced signaling has a positive feedback onto TGF-β1 transcription. Thereby TGF-β1 neutralization might additionally reduce TGF-β1 expression in CARL-mutant driven cells.

We found increased Nuclear factor kappa-light-chain-enhancer of activated B cells (NF-κB) RNA and NFκB related RNA expression in 32D-MPL-CALR^del52^ cells compared to 32D-MPL-CALR^WT^ cells (Supplementary Figure S9A). However, there was only a trend towards increased total NFκB protein expression, while there was no increase of NFκB phosphorylation and inhibitor of NF-κB (IκB) degradation in 32D-MPL-CALR^del52^ cells compared to CALR^WT^ (Supplementary Figure S9B-D). We observed that exposure of 32D-MPL-CALR^del52^ cells to the NFκB inhibitor JSH-23 did not cause a change in TNF-α production (Supplementary Figure S9E). These data indicate that oncogenic CALR^del52^ leads to increased NFκB RNA expression but not increased NFκB activation. Additionally NFκB activation is not required for TNF-α production.

**Figure 6:**
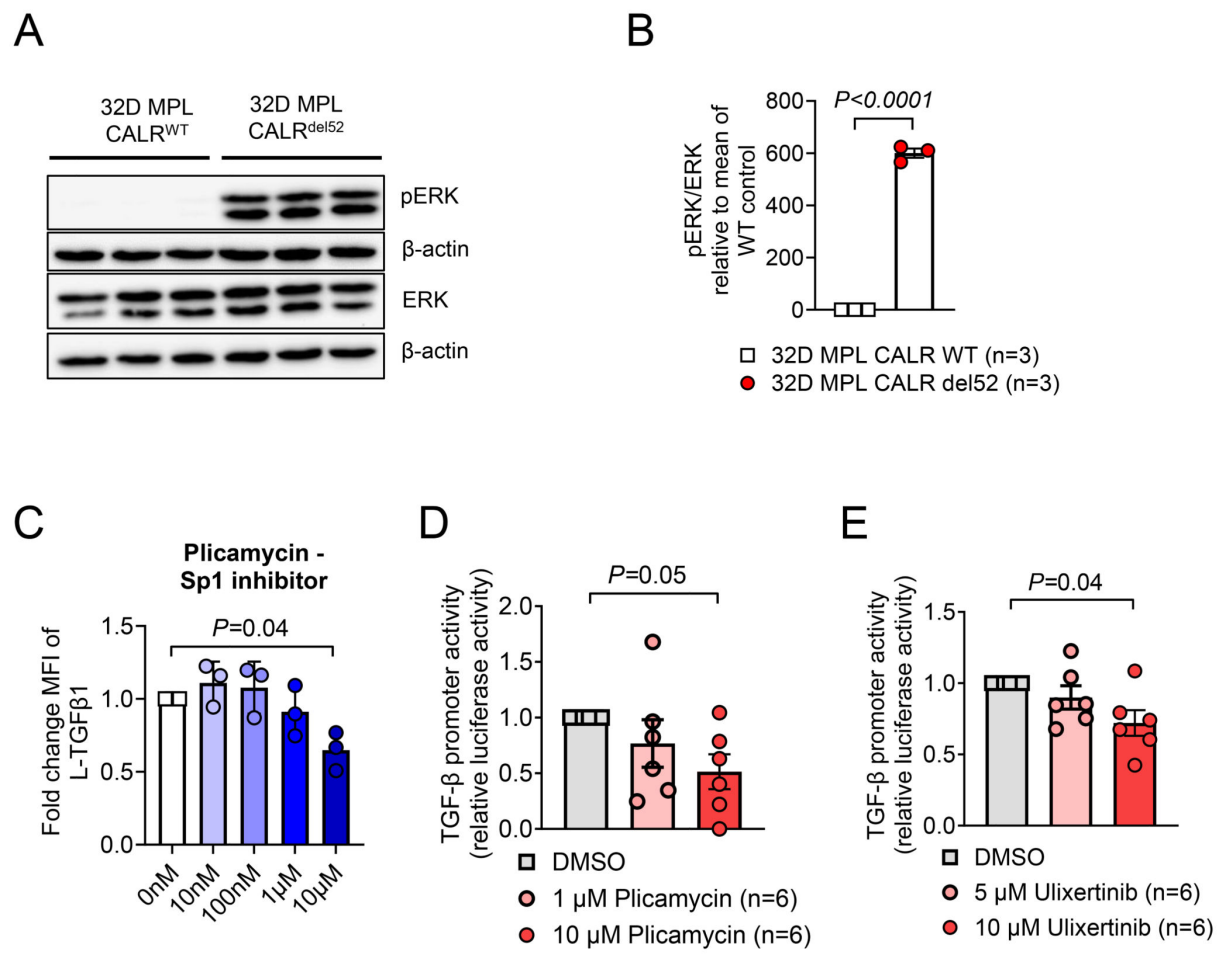
TGF-β expression is regulated by an ERK/Sp1 dependent pathway **(A, B)** Representative western blot (A) and relative quantification of phosph-ERK / total-ERK (B). Protein was isolated from 32D-MPL-CALR^WT^ or -CALR^del52^ after overnight IL3 starvation Pooled data from three independent experiments. *P*-values were calculated using unpaired Student’s *t test*. **(C)** Scatter plot showing expression of L-TGFβ1 on 32D-MPL-Calr^del52^ cells after overnight treatment with the Sp1 specific inhibitor (Plicamycin) and IL-3 withdrawal. *P* values were calculated using ordinary one-way ANOVA. **(D)** Scatter plot showing luciferase activity in 32D-MPL-Calr^del52^ cells, transfected with pGL3-TGFβ1 promotor vector, treated overnight with indicated concentrations of Sp1 inhibitor (Plicamycin) after IL-3 withdrawal. Pooled data from two independent experiments. P values were calculated using RM one-way ANOVA. **(E)** Scatter plot showing luciferase activity in 32D-MPL-Calr^del52^ cells, transfected with pGL3-TGFβ1 promotor vector, treated overnight with indicated concentrations of ERK inhibitor (Ulixertinib) after IL-3 withdrawal. Pooled data from two independent experiments. P values were calculated using RM one-way ANOVA. Bar plots show mean with SEM.

Since ERK-inhibition reduced TGF-β production in CALR^del52^ cells we next analyzed the role of ERK. Introduction of CALR^del52^ into 32D cells led to an increase of ERK activation (Figure 6A, B). To identify the transcriptional mechanism of how TGF-β is upregulated we performed inhibition of the ERK downstream transcription factor Sp1, using plicamycin. It was previously shown that Sp1 phosphorylation by ERK stimulates DNA binding (40). When exposing 32D-MPL-CALR^del52^ cells to plicamycin, we observed reduced TGF-β production (Figure 6C). TGF-β promoter activity in CALR^del52^ cells was reduced by plicamycin exposure (Figure 6D). In line with the central role of the CALR-del52/ERK/Sp1 axis for TGF-β transcription we detected reduced TGF-β promotor activity in 32D-MPL-CALR^del52^ when treated with ERK inhibitor Ulixertinib (Figure 6E). Besides Sp1, we also tested the roles of other ERK and PI3K downstream target transcriptions factors. As possible targets we analyzed AP-1, Elk-1, CREB, Foxo1 and NF-κB (Supplementary Figure S10A-E). We used inhibitory molecules for these transcription factors to treat 32D-MPL-CALR^del52^ cells and analyzed TGF-β expression. In contrast to Sp1 inhibition, none of these inhibitors blocked TGF-β expression (Supplementary Figure S10A-E).

These findings indicate the central role of the JAK1/2/ERK/Sp1 axis for TGF-β transcription in CALR^del52^ cells.

### BM cells derived from patients with myeloproliferative neoplasms exhibit increased TGF-β expression

**Figure 7:**
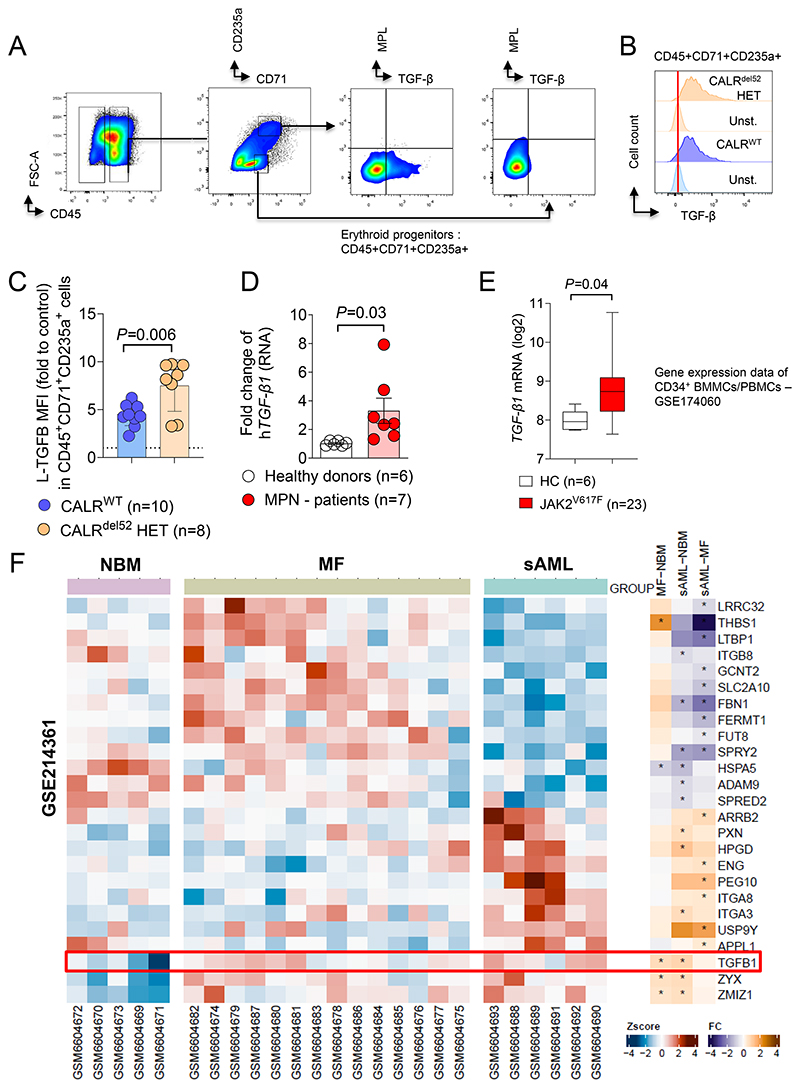
TGF-β1 expression is increased in patient derived MPN samples **(A)** Gating strategy for TGF-β and MPL quantification on CALR^del52^ or CALR^WT^ iPSC-derived erythroid progenitor cells CD45^+^CD71^+^CD235a^+/-^. **(B)** Representative histogram plot showing TGF-β expression on the surface of CALR^del52^ or CALR^WT^ iPSC-derived CD45^+^CD71^+^CD235^+^ erythroid progenitors with respective unstained control. **(C)** Scatter plot showing relative MFI of L-TGF-β surface expression on CALR^WT^ or CALR^DEL52^ iPSC-derived CD45^+^CD71^+^CD235a^+^ erythroid progenitor cells, fold change to MFI of unstained control. CALR^DEL52^ iPSC and CRISPR repaired CALR^WT^ control were generated from one CALR^del52^ MPN patient. Each dot represents individual experiment. The P-value was calculated using unpaired Student´s t test. **(D)** Scatter plot showing fold change RNA expression of human TGF-β1 in total mononuclear BM cells from MPN patients or healthy donors from the Freiburg MPN cohort. **(E)** Box plot showing TGF-β1 RNA expression (log2) in CD34^+^BMMCs/PBMCs from publicly available data set (GSE174060 - (42)). **(F)** Heat map showing expression profiling by GeneChipHuman Gene 2.0 ST Arrays (Affymetrix) of Lin^-^CD34^+^ PBMCs of normal bone marrow donors (NBM), patients with primary myelofibrosis (MF) and secondary AML (sAML). Data were obtained and re-analyzed from Gene Expression Omnibus (GEO) Database - GSE214361 (43). A set of genes involved in TGF-β1 regulation and activation was selected. Z score for separate patients is shown on the left and fold change comparing each group is shown on the right (* indicates significant changes).

To validate the findings made in mice, we used a human patient derived iPSC cell system that was previously described (18). We observed that MPL is expressed in human erythroid progenitors (Supplementary Figure S11A, B), which corresponds to our mouse system, where MPL is introduced together with CALR^DEL52^. MPL expression was higher in erythroid progenitors isolated from CALR^DEL52^ MPN patient-derived iPSCs compared to WT iPSCs (Supplementary Figure S11A, B). TGF-β expression was higher in erythroid progenitors isolated from CALR^DEL52^ MPN patient-derived iPSCs compared to WT iPSCs (Figure 7A-C).

Consistent with our finding others have shown that human CD71^+^ erythroid cells suppress T cell activation (41).

In addition to the iPSC system, we analyzed TGF-β1 expression by qPCR in mononuclear cells isolated from patients diagnosed with MPN at the University Medical Center Freiburg. Supplementary Table S1 provides the characteristics of all analysed patients. We included JAK2^V617F^ driven MPNs because the JAK2 downstream signaling is similar to patients with an oncogenic CALR mutation. 3 of 7 patients had a CALR-del52 mutant MPN. Mononuclear BM cells derived from MPN patients exhibited increased TGF-β1 expression on the RNA level compared to healthy donors (Figure 7D). To validate the results in an additional patient cohort, we analyzed publicly accessible data sets (GSE174060 and GSE214361). The study by Baumeister et al. investigated the expression profile of CD34^+^ bone marrow mononuclear cells / peripheral blood mononuclear cells (GSE174060) (42). Patients carrying JAK2^V617F^ mutation exhibited a higher expression of TGF-β1 compared to healthy donor cells (Figure 7E). Furthermore, expression of downstream TGF-β1 signaling genes was elevated in patient cells. Kong et al. performed gene expression profiling by high-throughput sequencing in healthy donor (NMB), myelofibrosis (MF) and secondary AML (MPN derived) patient cells (GSE214361) (43). We analyzed the microarray data set (GSE214361) for gene expression related to TGF-β. TGF-β1 expression was higher in BM cells derived from MF patients compared to healthy donors (NMB) (Figure 7F). Also TGF-β1 expression was higher in MPN-derived sAML BM samples compared to BM derived from healthy donors (Figure 7F). In line with these findings, we found that MF patient-derived BM cells exhibited increased expression of genes encoding proteins involved in regulation of TGF-β activation (*THBS1*) and co-activators for TGF-β signaling (*ZMIZ1*) (Figure 7F). Thrombospondin-1 (THBS1) is a key regulator for the activation of latent TGF-β (44). We observed that genes encoding proteins involved in TGF-β surface expression such as *LRRC32* (45,46), *LTBP1* (47) and *THBS1* were downregulated in sAML derived patient cells and genes encoding for negative regulators of TGF-β signaling such as *PEG10* (48) were upregulated when compared to MF-derived patient cells (Figure 7F). Using the same data set we performed progeny pathway annotation analysis (Supplementary Figure S12). TGF-β pathway activity was increased in BM derived from MF patients compared to BM derived from healthy donors (Supplementary Figure S12A).

**Figure 8:**
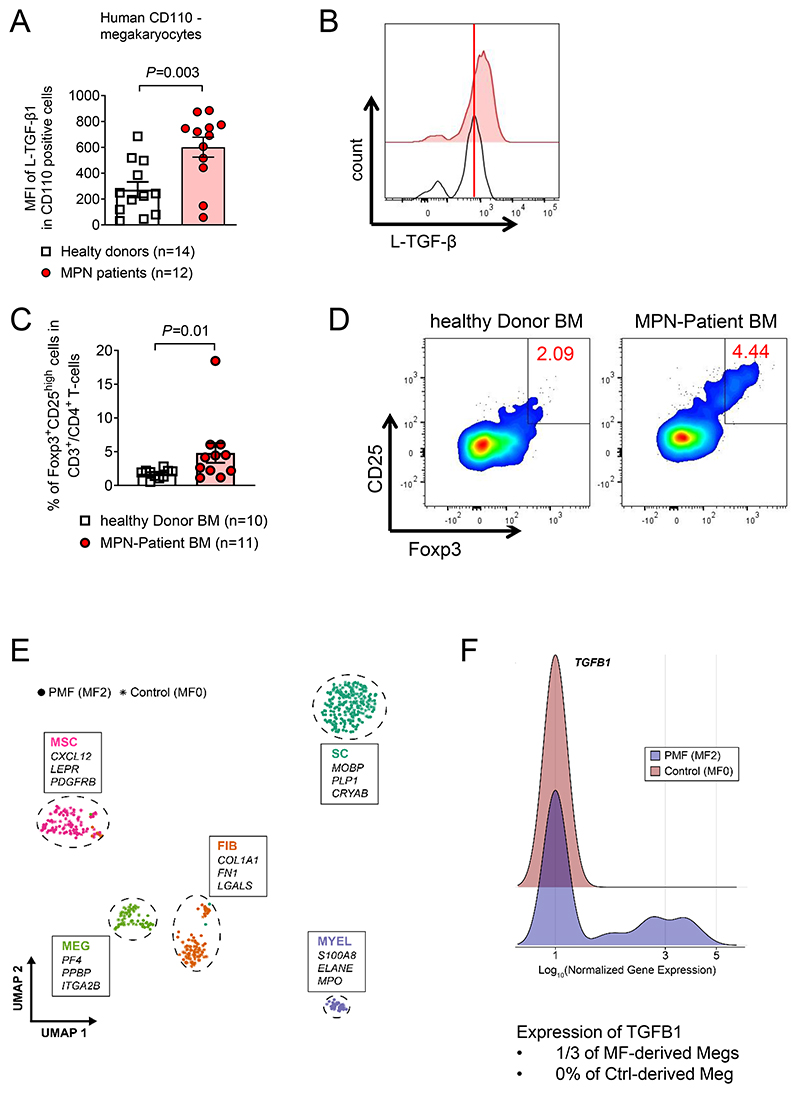
Cell types expressing TGF-β1 and T_regs_ frequencies in patient derived MPN samples **(A-B)** Scatter plot (**A**) and representative histogram (**B**) showing expression of L-TGF-β1 on CD45^+^CD110^+^ BMMNCs of healthy donors or MPN patients (Freiburg MPN cohort). *P*-values were calculated using unpaired Student’s t-test **(C, D)** Scatter plot (**C**) and representative flow cytometry plots (**D**) showing percentage of CD25^high^Foxp3^+^ T_reg_ cells in CD3^+^CD4^+^ BMMNCs of healthy donors or MPN patients (Freiburg MPN cohort). *P*-values were calculated using unpaired Student’s t-test. **(E, F)** UMAP (E) of publicly available dataset (GSE156644). Niche-derived single-cell transcriptomes of 1 JAK2^V617F^-positive PMF-patient (MF 2-3) and two control patients (MF 0). Differential expression of marker genes between clusters was used to verify cellular identity. Top 3 leading marker genes based on cluster-wise average expression and biological function are highlighted. Ridgeline plot (F) depicting the distribution of log-normalized gene expression for TGFB1 within the megakaryocyte cluster (MEG) after subsetting. Probability density is shown comparing cells derived from myelofibrotic (blue) and non-fibrotic BM (red).

To more specifically determine the cell types that produce TGF-β, human mononuclear cells were isolated and stained for CD110 as a megakaryocyte lineage marker (Supplementary Figure S13A). CD110^+^ cells isolated from the BM of MPN patients exhibited increased TGF-β1 protein levels compared to healthy donor-derived mononuclear BM cells (Figure 8A-B). Since we had observed increased frequencies of T_reg_ cells in mice that received CALR^del52^MPL BM compared to control BM, we next analyzed T_reg_ frequencies in the Freiburg MPN cohort. We found an increased percentage of T_reg_ cells within all CD4^+^ T cells in the BM of MPN patients compared to healthy controls (Figure 8C, D, Supplementary Figure S13B). These findings are in agreement with our observations made in the mouse model, were T_reg_ were increased in mice carrying CALR^del52^ BM.

Using scRNA seq of an additional MPN patient cohort, we found an increase of TGF-β1 RNA in human megakaryocytes derived from PMF patients compared to healthy controls (GSE156644) (Figure 8E-F). The patient cohort that was used for scRNA seq based analysis had been previously reported (25). The data indicate that in patients both CALR^del52^ and JAK2^V617F^ induce TGF-β expression in megakaryocytes in addition to myeloid cells and erythroid progenitor cells. Our findings are consistent with previous reports showing increased production of TGF-β by megakaryocytes and monocytes in patients with MPNs compared to healthy controls (49).

## Discussion

Clinical responses observed in MPN patients under treatment with interferon, indicate that these diseases are in principle susceptible to immunomodulatory therapy (50,51). Our study reports the functional connection between oncogenic calreticulin (CALR^del52^) and immunosuppressive TGF-β1 production in MPN which had not been described so far. Using scRNA-seq of primary mouse BM cells we observed that CALR^del52^ enhances TGF-β producing erythroid progenitor cells, which was in line with enhanced TGF-β production in 32D cells and Ba/F3 cells when oncogenic CALR was introduced. This is the first evidence for a functional contribution of oncogenic CALR to TGF-β mediated immune escape, which could be responsible for the resistance of MPN towards certain immunotherapies.

At the mechanistic level we delineate the molecular signaling pathways driving CALR-induced TGF-β production (Supplementary Figure S14). TGF-β1 production by CALR^del52^ cells was dependent on JAK1/2, PI3K and ERK activity, thereby identifying these kinases as potential therapeutic targets to counteract the immunosuppressive effects of TGF-β in CALR^del52^ MPN. Conversely, we found no role of mTOR or STAT3 activation for TGF-β-production.

Our finding that the CALR^del52^ / ERK / Sp1 axis induces TGF-beta production in MPN is novel. The role of Sp1 we found here is in agreement with prior work showing that Smad–Sp1 complexes mediate TGFβ-induced transcription of oncogenic Smad7 (52). Additionally, Sp1 and Smad transcription factors co-operate to mediate TGF-beta-dependent activation of amyloid-beta precursor gene transcription (53).

Furthermore, we were able to characterize the immunoregulatory effects of TGF-β in MPN. We found that TGF-β inhibition with an anti-TGF-β antibody induced anti-MPN activity *in vivo*, while T cell depletion ablated the protective effects of neutralizing TGF-β. Targeting TGF-β *in vivo* increased glycolytic activity and TNF-α production of CD4^+^ T cells. TGF-β exposure reduced cytotoxic perforin and TNF-α production by T cells *in vitro*.

TGF-β has been understood to suppress the functions of effector T cells and ablation of TGF-β signaling in T cells caused multiorgan autoimmunity in mice (54), while inhibition or deletion of TGF-β activated kinase-1 reduces inflammation (55). However, recent reports suggest that TGF-β superfamily members enhance IFN-γ production by effector/memory CD8^+^ T cells (56). Additionally cytotoxic T cells isolated from healthy donors and cancer patients eliminate cancer cells in a TGFβ-dependent manner (57).

The BM microenvironment undergoes massive alteration during MPN progression including a shift into a pro-inflammatory milieu due to increased production of many immune-modulatory cytokines (58). We confirmed differential expression of chemoattractant cytokines including Cxcl2 and Cxcl3 in addition to increased expression of pro-inflammatory cytokines including TNF-α and immune regulatory cytokines like TGF-β1, IL-4 and IL-15 in our CALR dependent MPN model. These changes in expression were driven by MPL signaling induced by oncogenic CALR. Our finding that CALR causes TGF-β expression may be counter intuitive as TGF-β can have an inhibitory effect on tumor cell proliferation (59), but this is counteracted in our MPN cells by downregulation of key TGF-β signaling mediators like Tgfbr1, Tgfbr2, Smad1 and Smad4 together with simultaneous upregulation of SMAD Specific E3 Ubiquitin Protein Ligase 1 (Smurf1) responsible for regulating proteasomal degradation of SMAD1 and SMAD5. In the past years, TGF-β-signaling has been identified as an important therapeutic target in tumor therapy (60,61). The broad effect of TGF-β on regulation of immune surveillance (62) and epithelial–mesenchymal transition (63) makes this cytokine a key regulator in tumorigenesis. We performed *in vivo* TGF-β neutralization using anti-TGF-β antibody in our MPN mouse model to study its effect on disease progression and the immune system. We found a survival benefit for mice treated with a neutralizing anti-TGF-β antibody. T cells isolated from these mice exhibited an overall increase in glycolytic capacity. This indicates a positive effect of TGF-β neutralization on T cell metabolism and cytokine production. To evaluate the functional role of T cells, we performed TGF-β neutralization in T cell-depleted mice. T cell depletion alone was sufficient to reverse the survival benefit achieved by TGF-β neutralization. To further test this, we used TGF-β neutralizing antibodies in a post allogeneic transplantation model using the transformed cell line 32D-MPL-CALR^del52^. This model relies on the graft versus leukemia effect (GvL) induced by histocompatibility complexes on the 32D cells recognized by the allogeneic T cells. By utilizing this, we can analyze the effect of the treatment on T cell/sAML-blast interaction. Anti-TGFβ treatment improved survival of MPN bearing mice compared to the allo-Tc + isotype group supporting the concept that the CALR^del52^ / TGF-β axis reduces the GvL effect. To test the impact of TGF-β on T cells in a well-controlled system, we used *in vitro* T cell activation using CD3/CD28 activator beads. TGF-β reduced perforin and TNF-α production by T cells and induced the expansion of T_reg_, which is consistent with previous reports showing that TGF-β can induce T_reg_ cells (64), a cell type with potent immunosuppressive function (65). These *in vitro* findings and the increased numbers of T_reg_ cells in MPN mice indicate the central role of TGF-β in immune evasion during MPN progression. Previous studies have shown that CALR and JAK2^V617F^ specific T cells exist in patients with MPNs and that vaccination against mutant CALR in MPN causes the increase of CALR specific T cells (66). Additionally, it was shown that Immune checkpoint blockade causes an increase of T-cell immunity against CALR^del52^ (67). Moreover, U2AF1 neoantigens were found in human MPN (68). It is conceivable that TGF-β and the T_reg_ cells induced by this cytokine reduce the activity of anti-MPN directed T cells.

We observed a connection between JAK/STAT, ERK/Sp1 and PI3K signaling with respect to induction of TGF-β expression. Previous studies revealed a compensatory role of MEK/ERK signaling following ruxolitinib treatment in JAK2^V617F^ driven MPN (69,70). Combination treatment of ruxolitinib and MEK-inhibitor had a synergistic therapeutic effect in MPN patients, lowering the numbers of progenitor cells (70,71). Besides the direct inhibitory effects of the JAK1/2 plus MEK inhibitor combination on cell proliferation, the synergism may be due to reduced TGF-β production. Our previous work had shown that inhibition of JAK/STAT reduces cytokine production (72) and acute GVHD (73).

To understand the clinical relevance of our findings we examined BM cells derived from MPN patients. We included JAK2^V617F^ driven MPNs because the JAK2 downstream signaling is similar to patients with an oncogenic CALR mutation. We observed increased L-TGF-β1 expression in MPL (CD110) positive BM cells of patients from our Freiburg MPN cohort. We chose to analyze CD110 positive cells, as a *CALR* mutation was shown to be functionally active in this sub-population (74). To validate these findings in independent patient cohorts, we analyzed publicly available data sets of MPN patients. The studies by Baumeister et al. and Kong et al. both provided gene expression data from CD34^+^ precursor cell populations derived from the BM of healthy individuals, MPN patients and patients with sAML derived from MPN (42,43). In both data sets we found increased TGF-β1 mRNA expression in the BM of MPN patients when compared to healthy controls. When comparing MPN and sAML we observed that expression of TGF-β regulatory proteins like THBS1, LRRC32 and LTBP1 was downregulated and expression of TGF-β signaling inhibitors, like PEG10, was upregulated in sAML patients. This might indicate a shift in responsiveness towards TGF-β. A key feature in transition from MPN to sAML is the development of a blast phase accompanied by clonal cell expansion. TGF-β as a driver of differentiation may be inhibited by PEG10, when MPN progresses towards the blast phase with an increase of non-differentiated cells.

In summary, we have delineated that the CALR^del52^-TGF-β1 axis causes decreased T cell activity and glycolytic capacity. Mechanistically, we could show that TGF-β1 production is dependent on JAK1/2, PI3K, ERK and Sp1 activity in CALR^del52^-mutant cells (Supplementary Figure S 14). These findings reinforce the rationale for conducting clinical trials to explore the therapeutic potential of TGF-β antagonists in MPN patients.

## Supplementary Material

Supplementary Material

## Significance of the findings in this research

Targeting the mutant calreticulin/TGF-β1 axis increases T cell activity and glycolytic capacity, providing the rationale for conducting clinical trials on TGF-β antagonists as an immunotherapeutic strategy in patients with myeloproliferative neoplasms.

## Abbreviations

AAO: amino acid oxidation
allo-HCT: Allogeneic hematopoietic cell transplantation
BM: bone marrow
BMP: Bone morphogenetic protein
CALR: Calreticulin
del: deletion
ERK: Extracellular signal-regulated kinases
ET: essential thrombocythemia
FAO: fatty acid oxidation
Foxp3: Forkhead-Box-Protein P3
GEO: Gene Expression Omnibus
GVHD: graft-versus-host disease
ins: insetion
IL: interleukin
iPSC: induced pluripotent stem cells
IκB: inhibitor of NF-κB
JAK: Januskinase
KI: knock-in
L-TGF-β1: latency-associated-peptide-TGF-β1
MF: primary myelofibrosis
MPL: thrombopoietin receptor
MPN: myeloproliferative neoplasms
mTOR: mammalian target of rapamycin
NF-κB: Nuclear factor kappa-light-chain-enhancer of activated B cells
OPP: O-Propargyl-Puromycin
PBS: phosphate buffered saline
PI3K: phosphatidylinositol 3-kinase
Plat-E: Platinum-E
Ruxo: Ruxolitinib
SCENITH: Single-cell energetic metabolism by profiling translation inhibition
scRNA seq: single-cell RNA sequencing
Sp1: specificity protein 1
STAT: signal transducer and activator of transcription
SCF: stem cell factor
TGF: Transforming growth factor
THBS1: Thrombospondin-1
TNF: Tumor necrosis factor
T_reg_: regulatory T cells
