# Supplementary material for: Oncogenic Calreticulin Induces Immune Escape by Stimulating TGF-β Expression and Regulatory T Cell Expansion in the Bone Marrow Microenvironment
Source: Cancer Res. Author manuscript; Available in PMC 2024 Sep 28. (PMC11405138; doi:10.1158/0008-5472.CAN-23-3553)
Supplement: Supplementary Material [file EMS198793-supplement-Supplementary_Material.pdf]

Supplementary Figure S1

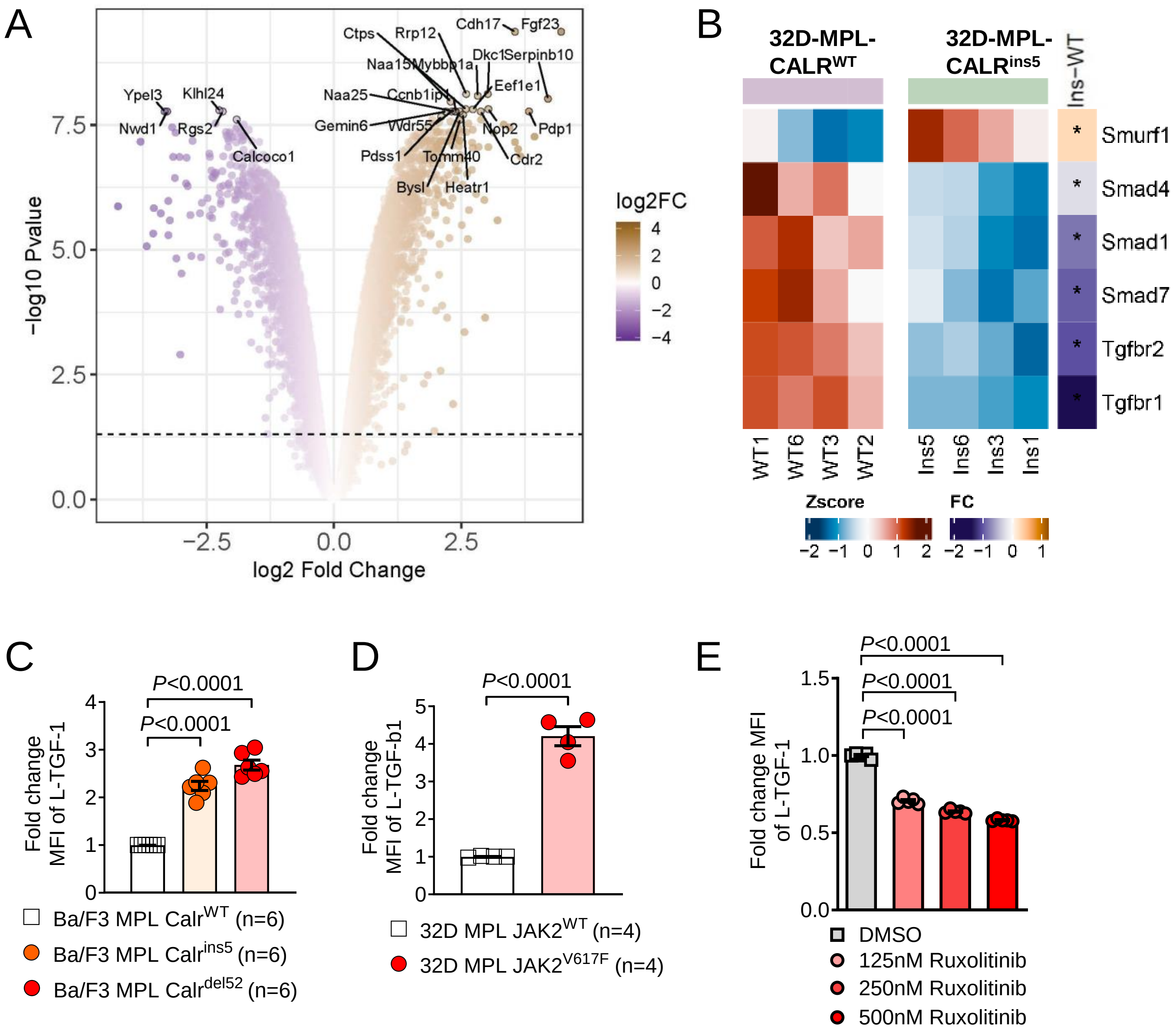

Supplementary Figure S2

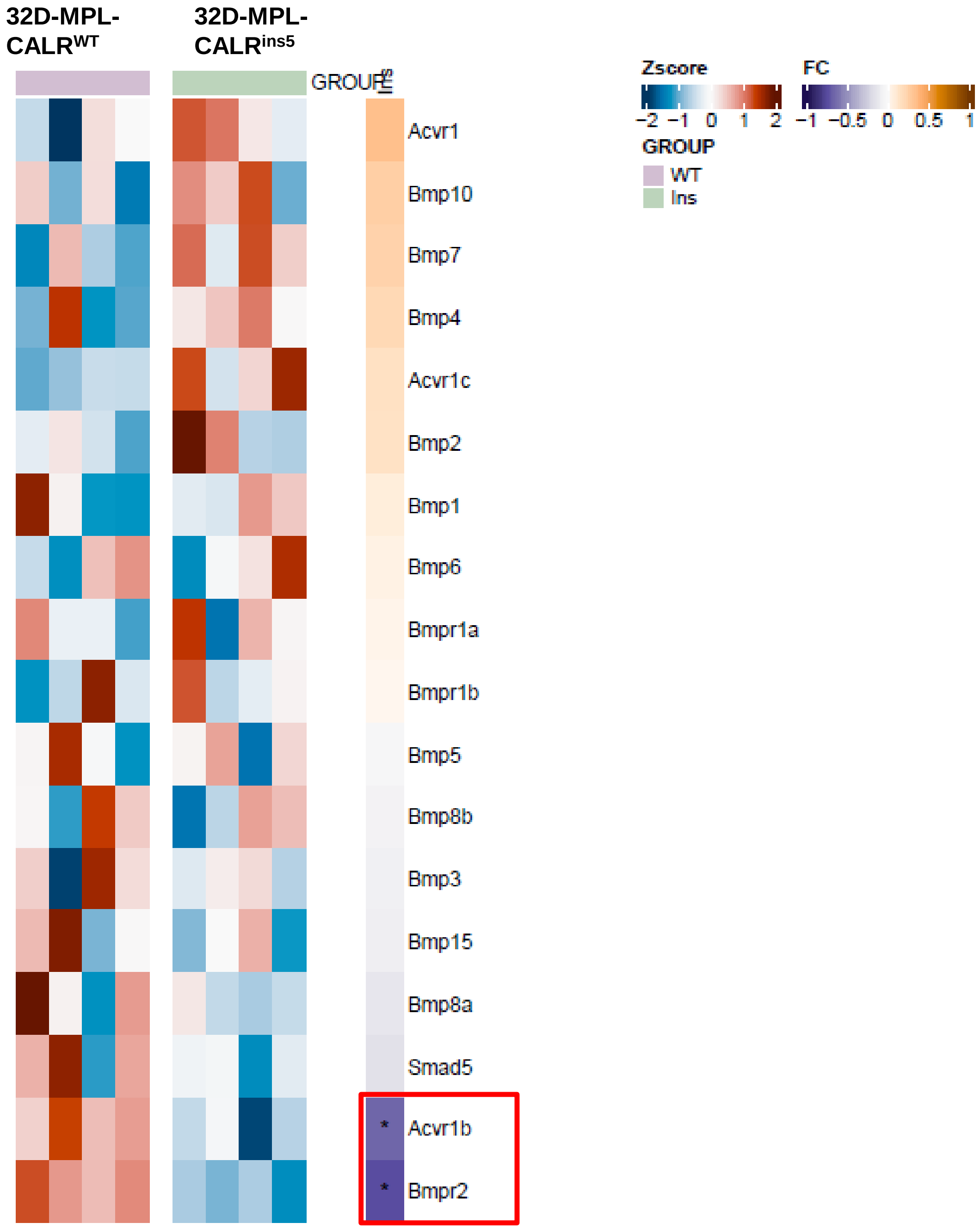

Supplementary Figure S3

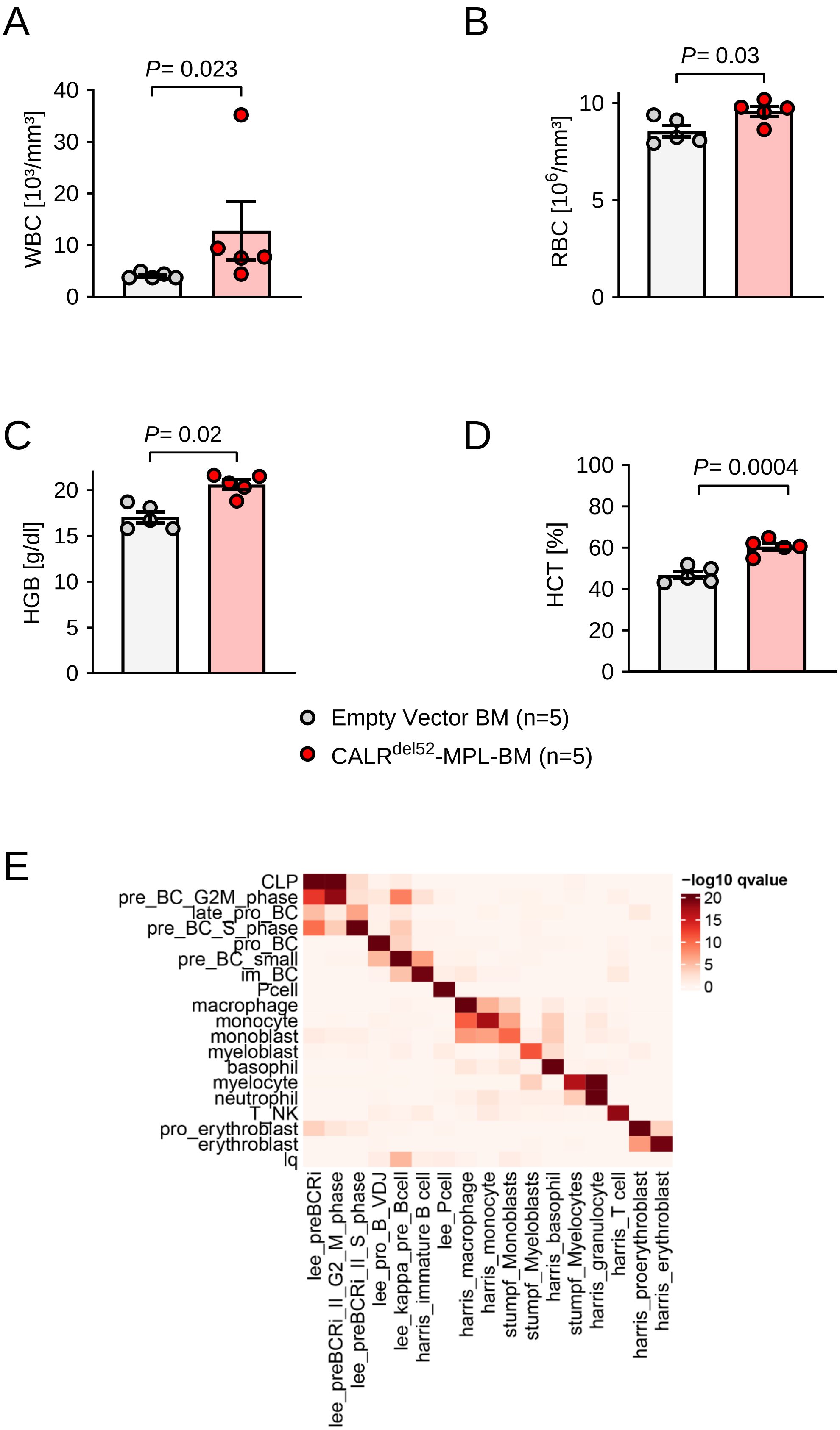

Supplementary Figure S4

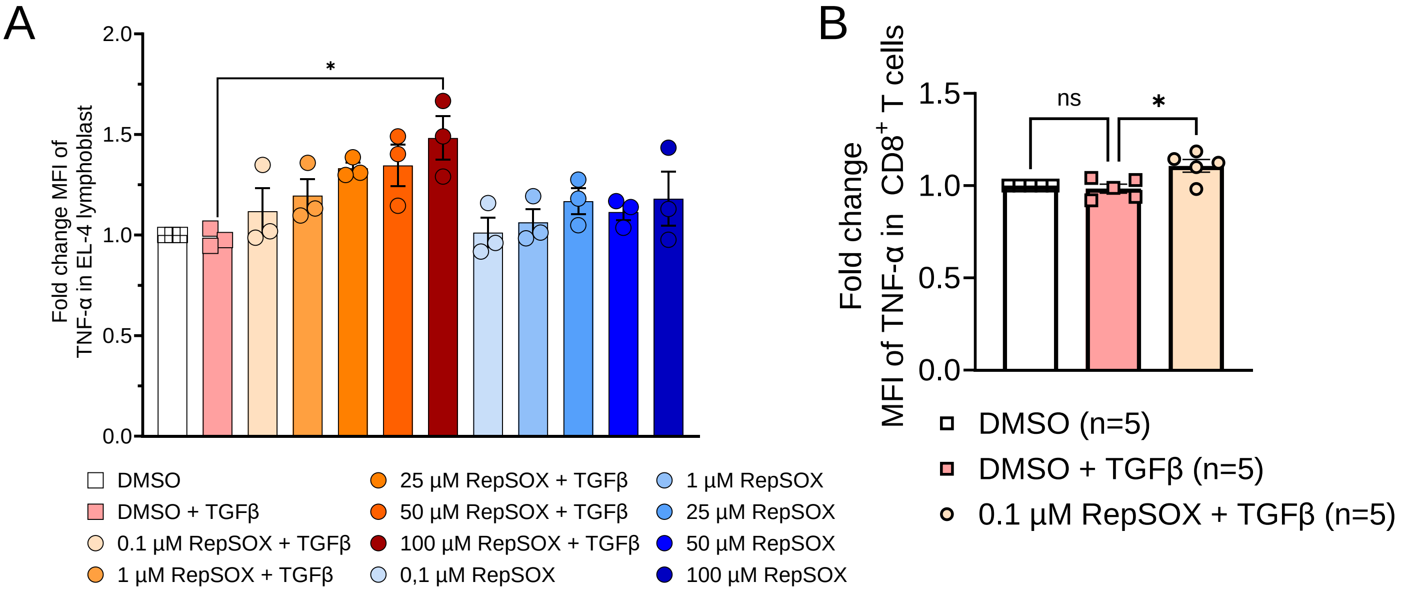

Supplementary Figure S5

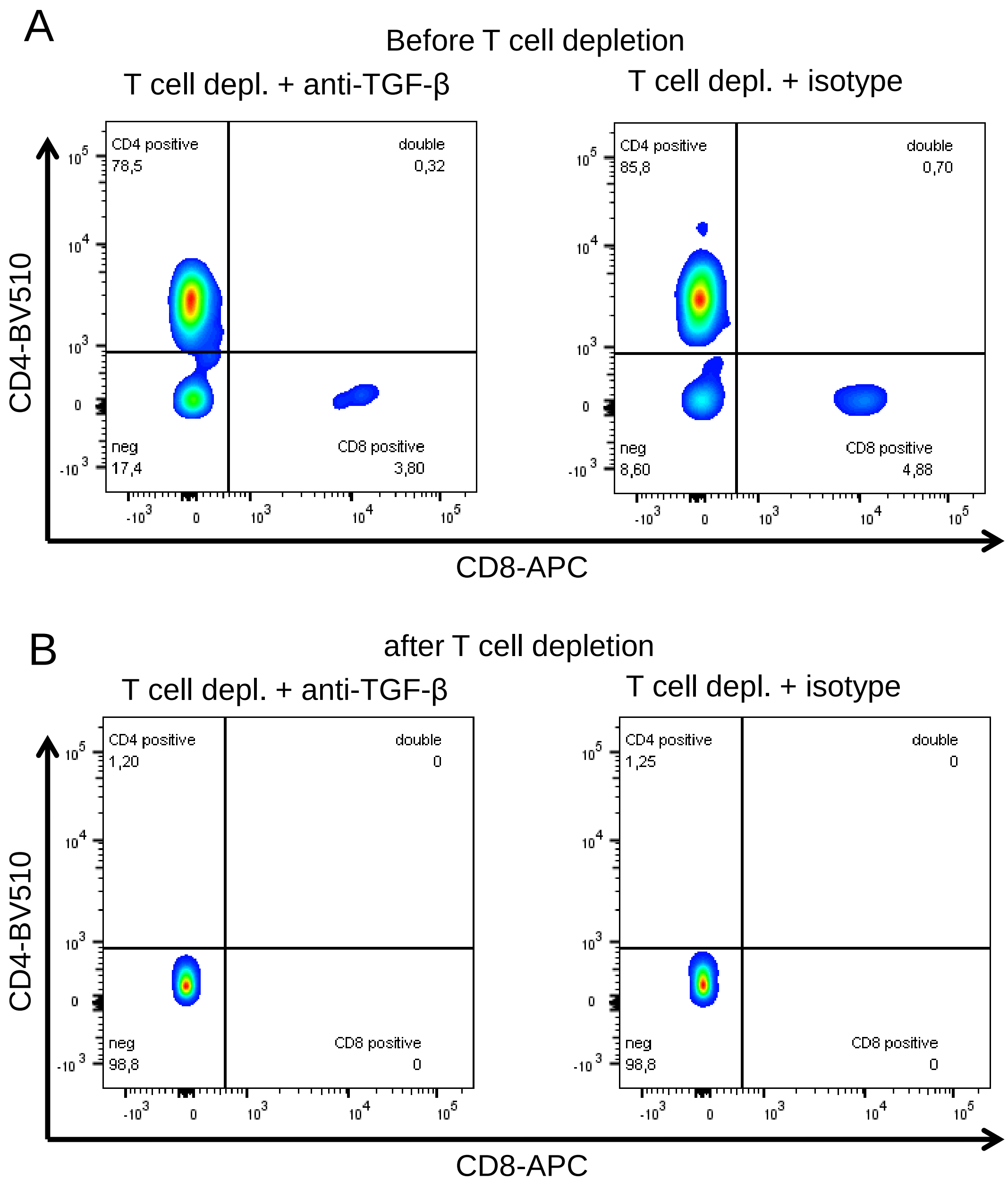

Supplementary Figure S6

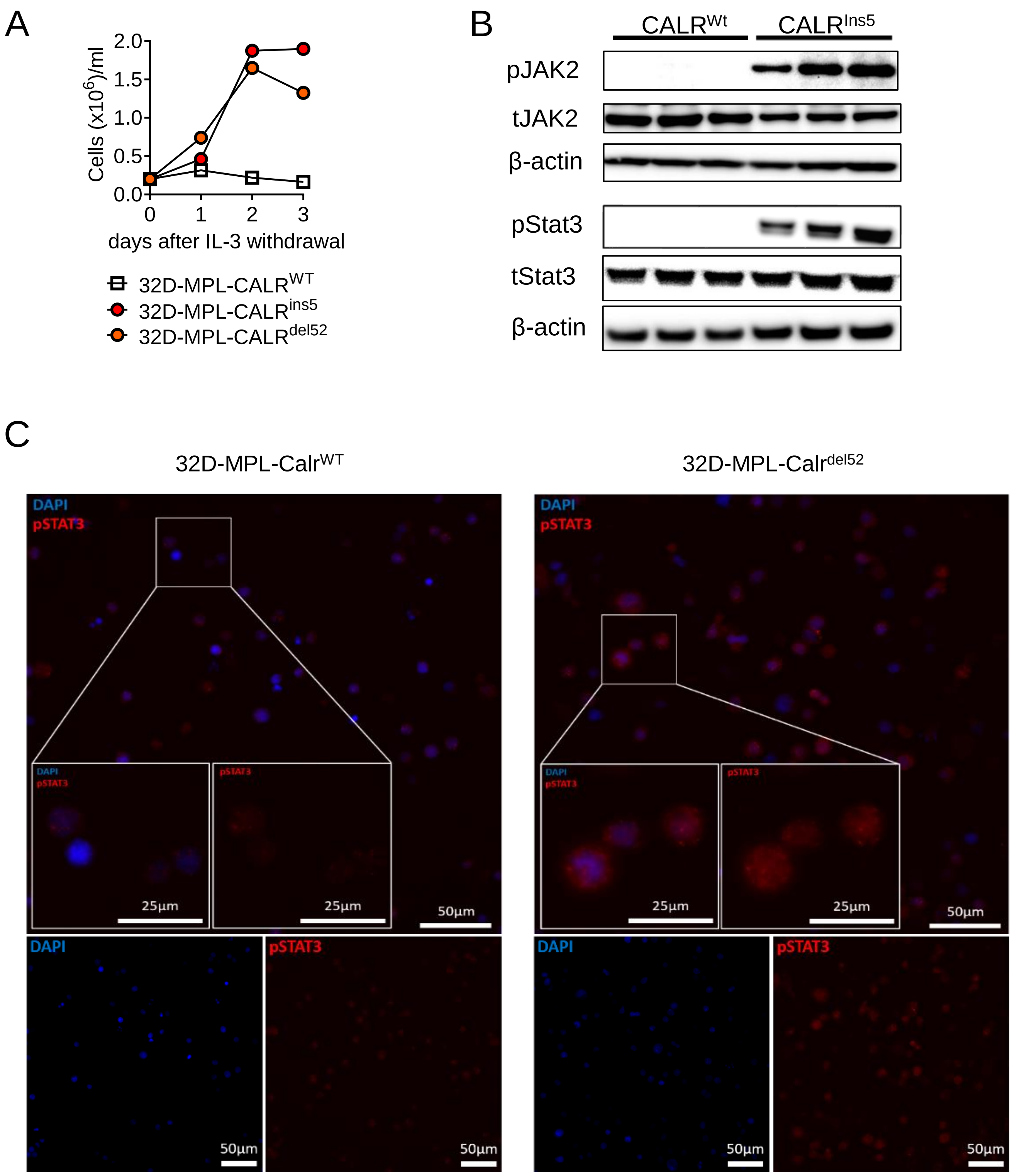

Supplementary Figure S7

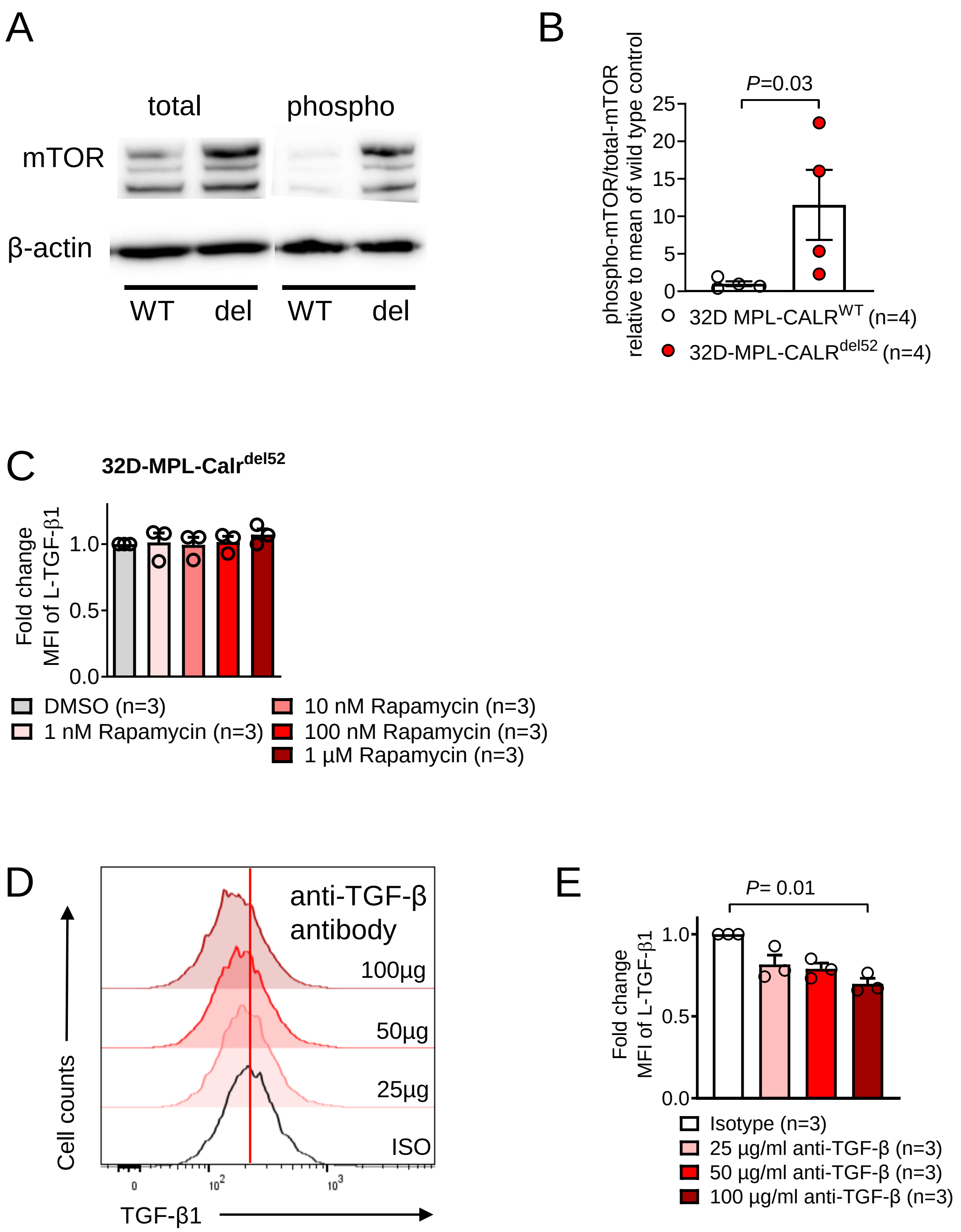

Supplementary Figure S8

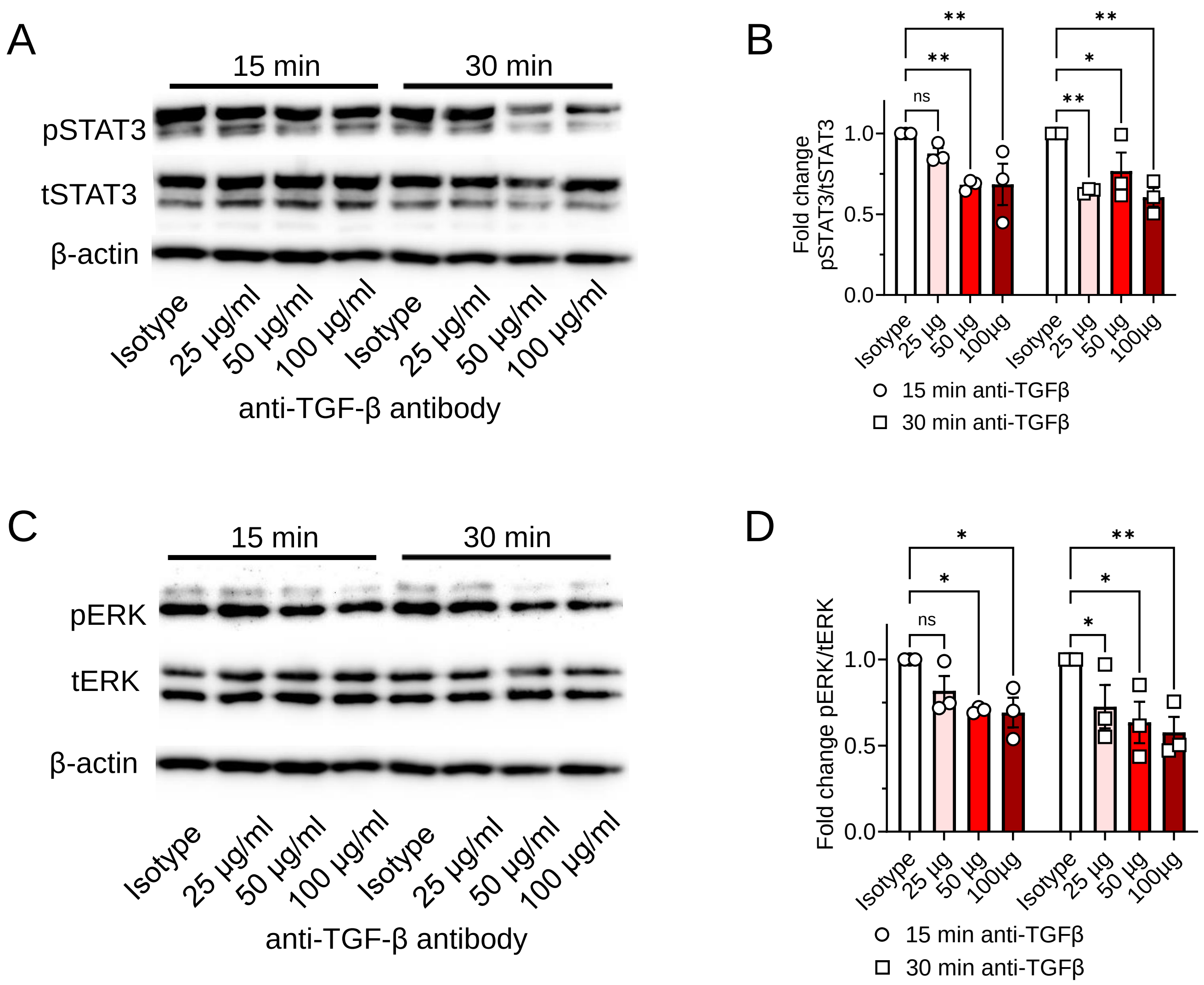

Supplementary Figure S9

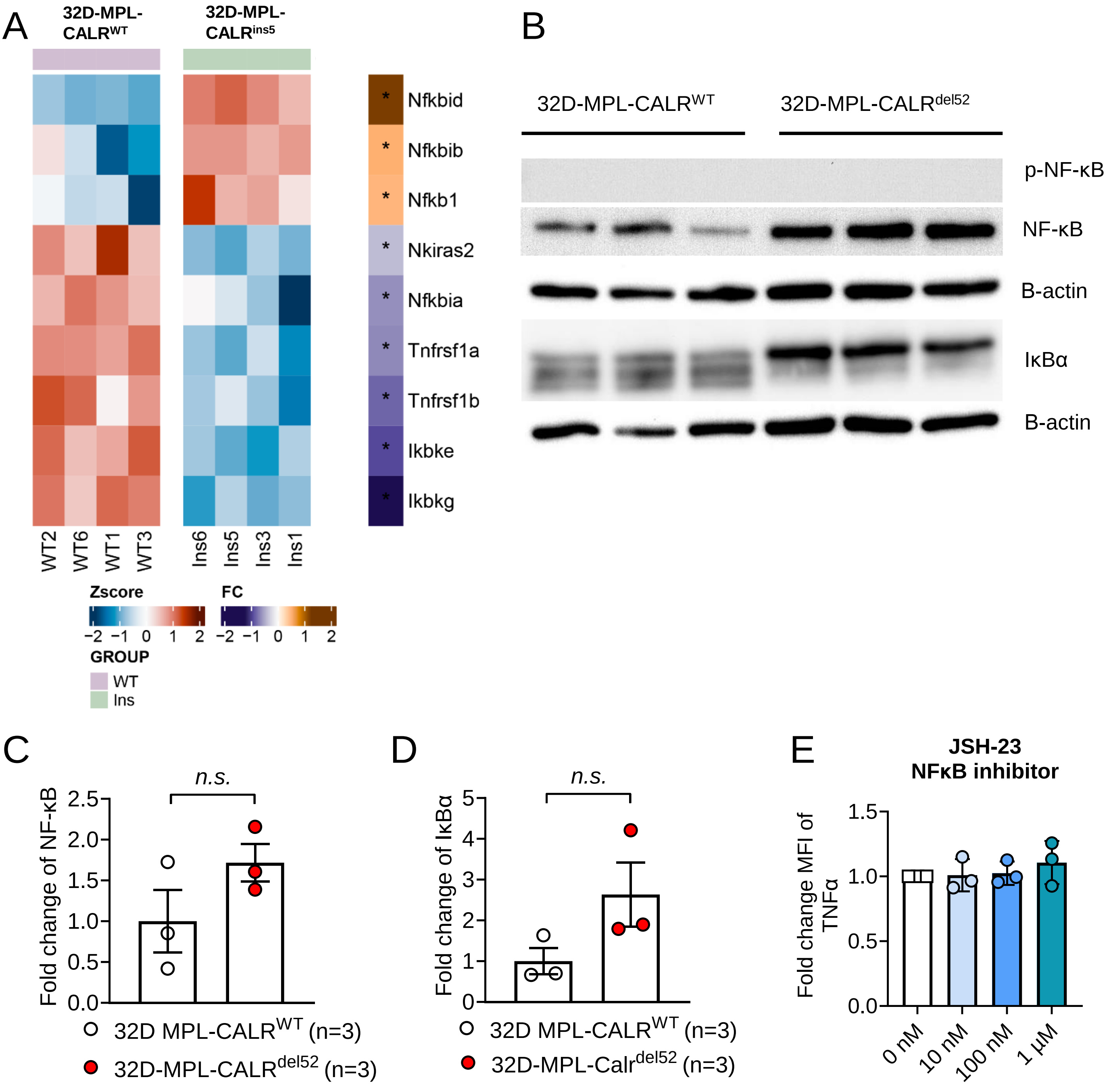

Supplementary Figure S10

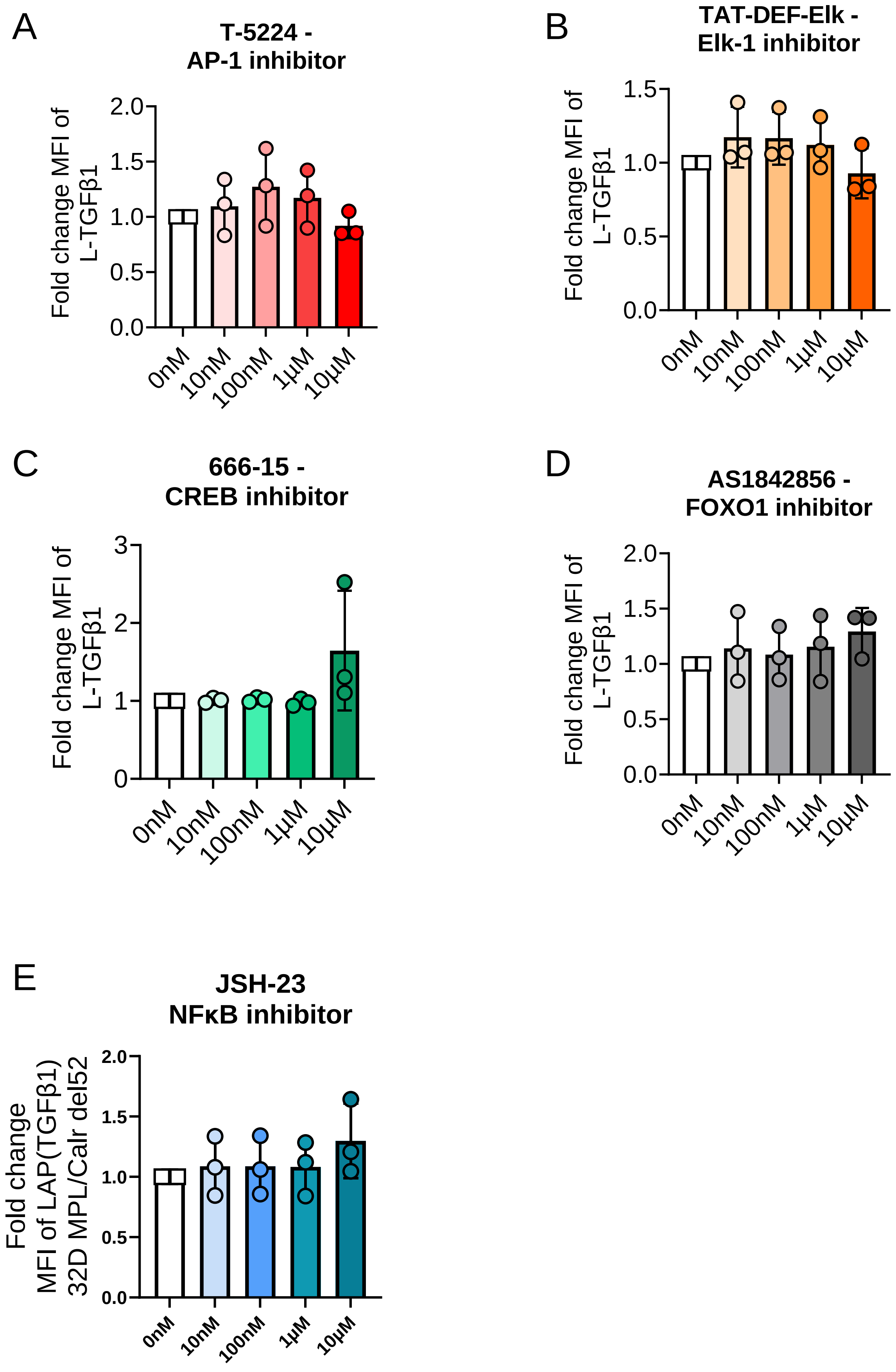

Supplementary Figure S11

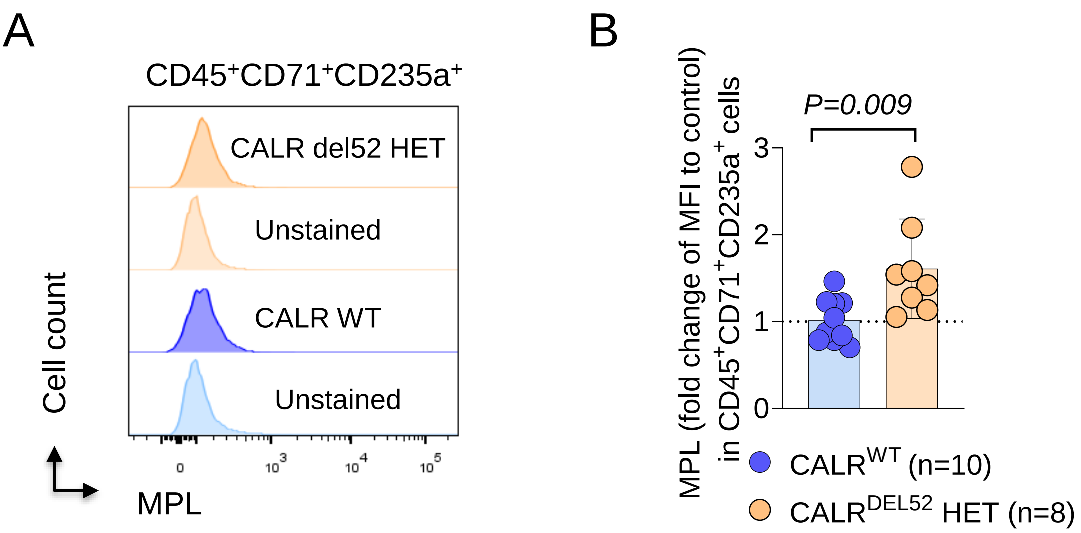

Supplementary Figure S12

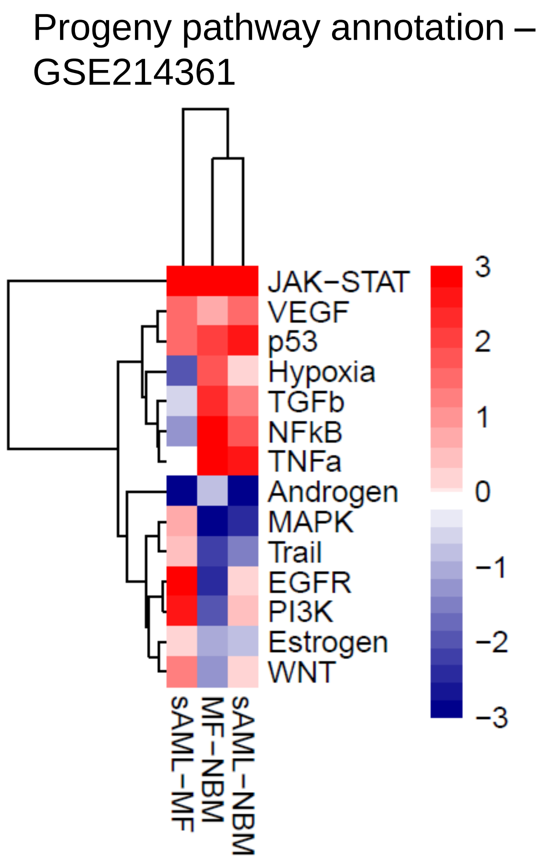

Supplementary Figure S13

Representative gating of patient bone marrow cells (Fig. 8A, B)

A

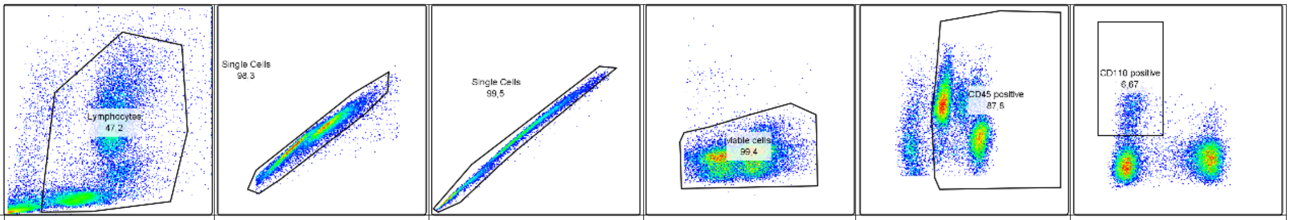

X-Axis	FSC-A	FSC-A	SSC-A	FSC-A	CD45-PB	CD15 FITC
Y-Axis	SSC-	FSC-H	SSC-H	Live/Dead (Zombie NIR)	FSC-A	CD110-APC

Representative gating of patient bone marrow cells (Fig. 8C, D)

B

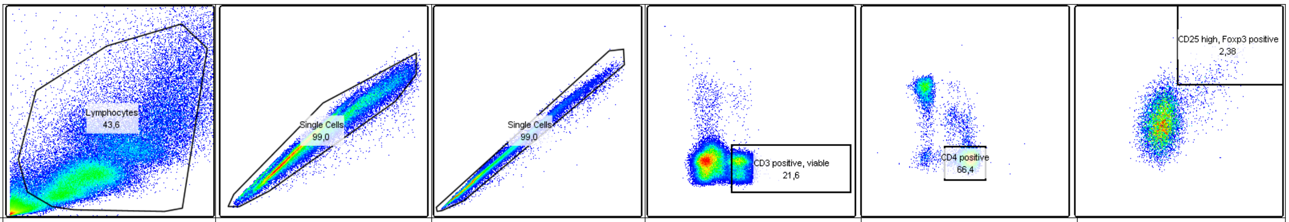

X-Axis	FSC-A	FSC-A	SSC-A	CD3-PB	CD4-BV510	FoxP3-PE
Y-Axis	SSC-	FSC-H	SSC-H	Live/Dead (Zombie NIR)	CD8-FITC	CD25-APC

Supplementary Figure S14

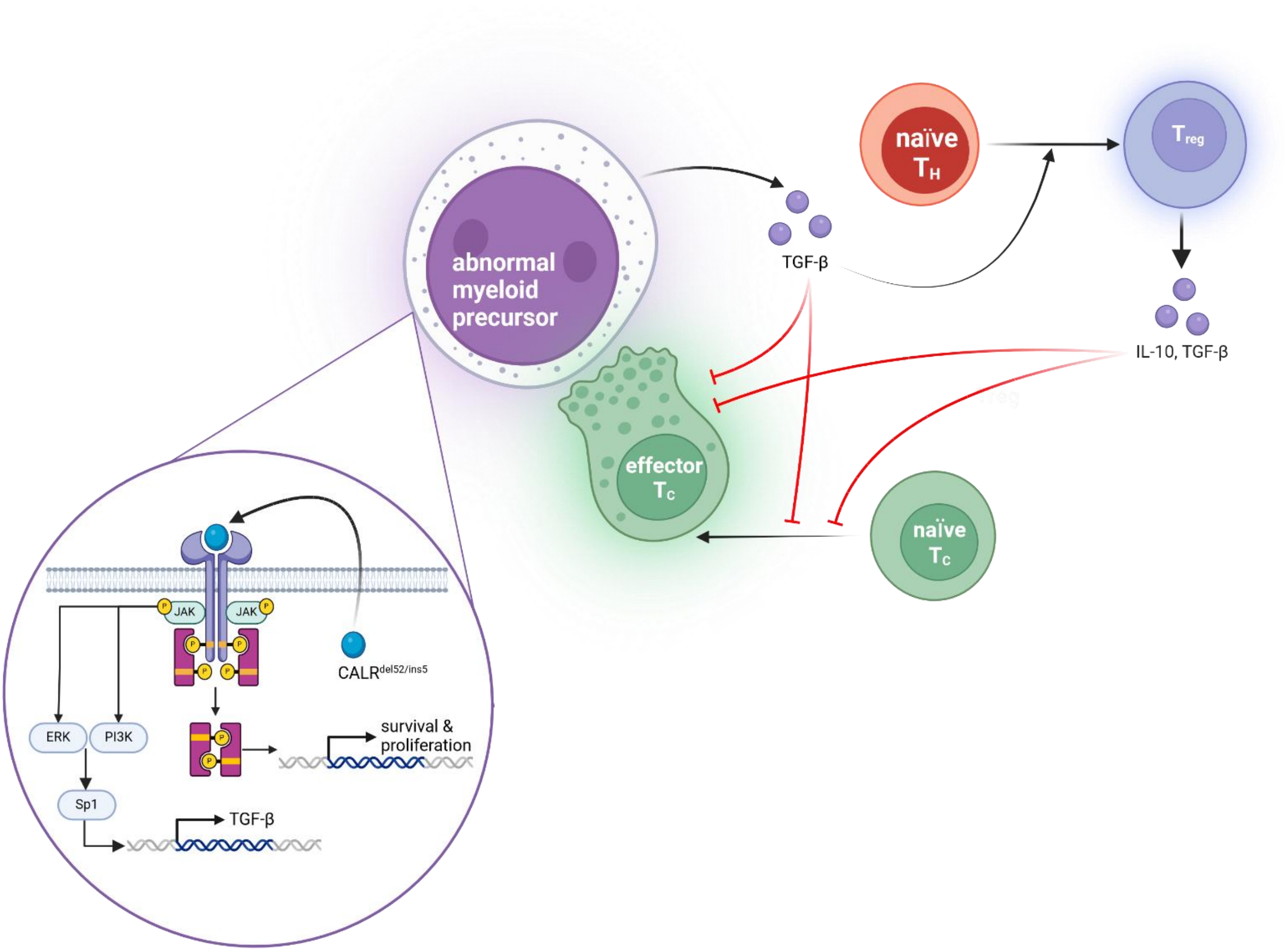

Supplementary Figure legends:

Supplementary Figure S1: Oncogenic JAK signaling induces TGF-β1 expression

(A, B) Volcano plot showing differentially regulated genes (A) and heat map showing relative expression of genes involved in TGF-β signaling (B) in 32D-MPL-CALR^{WT} cells compared to 32D-MPL-CALR^{ins5} cells. RNA was isolated from four independent IL3 starvations for each group. Differential gene expression analysis was performed with the linear model-based approach (limma R package). **(C-E)** Scatter plot showing fold change MFI of L-TGF-β1 on Ba/F3-MPL-CALR^{WT}, -CALR^{ins5},-CALR^{del52} cells (C); 32D-MPL-Jak2^{WT}, -Jak2^{V617F} cells (D); BA/F3-MPL-JAK2^{V617F} cells treated with indicated concentration of ruxolitinib overnight (E). Pooled data from six (C), four (D) and five (E) replicates. *P* values were calculated using ordinary one-way ANOVA (C, E) and unpaired Student's *t*-test (D).

Supplementary Figure S2: Bone morphogenetic protein (BMP) signaling molecules are not induced by oncogenic JAK signaling

Heat map showing expression profile of MPL-CALR$^{WT/ins5}$ transduced 32D cells. Relative expression of selective BMP signaling pathway molecules are shown. Color codes represents the Z-score log2 intensity. RNA was harvested from four independent IL3-starved cell cultures. Differential gene expression analysis was performed with the linear model-based approach (limma R package).

Supplementary Figure S3: Bone marrow transduced with CALR^{del52} and MPL induces MPN phenotype in mice

(A-D) Scatter plot showing white blood cell (WBC) count (A), red blood cell count (RBC) (B), hemoglobin concentration (HGB) (C) and hematocrit (HCT) (D) in mice on day 20 post injection of bone marrow cells transduced with empty vector control or CALR^{del52}-MPL. Data from one representative experiment. **(E)** Heat map depicting enrichments of cell type programs (= gene sets = top 50 DEGs per cell type cluster) (rows) with gene signatures (columns) previously defined for mouse bone marrow (1-3). Enrichments are calculated by hypergeometric test (-log10 FDR-adjusted P-values).

Supplementary Figure S4: TGF-β regulated TNFα expression in lymphoid cells

(A) Scatter plot showing TNF-α expression in the lymphoblast cell line EL-4 after 48h treatment with indicated concentration of TGFBR-1 inhibitor RepSOX and TGF-β (10 ng/ml) as indicated. **(B)** Scatter plot showing TNF-α expression in primary CD8^{+} T cells activated with CD3/CD28 activator beads for 72h and treated with TGFBR-1 inhibitor RepSOX (0.1 µM) and TGF-β (10 ng/ml), if indicated, for 24h.

Pooled date from three (A) or five (B) independent experiments. *P*-values were calculated using Friedman test.

Supplementary Figure S5: In vivo TGF-β neutralization improves survival of CALR^{del52} driven MPN

(A, B) Flow cytometry plots showing percentage of CD4-BV510 and CD8-APC positive cells in peripheral blood of CALR^{del52}-MPL-BM injected mice, before – d10 post BMT (A) and after – d53 post BMT (B) T cell depleting antibody therapy.

Supplementary Figure S6: validation of oncogenic CALR^{del52} induced downstream signaling

(A) XY plot showing density (*10^{6} cells/ml, Y-axis) of 32D-MPL-Calr^{WT}, -Calr^{ins5} and -Calr^{del52} cells over time (days, X-axis) after IL-3 withdrawal.

(B) Western blot showing total-/phospho-JAK2 and total-/phospho-STAT3 with respective β-actin, comparing 32D-MPL-CALR^{WT} with 32D-MPL-CALR^{del52} cells after overnight IL3 withdrawal. Protein isolated from three independent IL-3 starvations.

(C) Representative fluorescence microscopy pictures showing 32D-MPL-CALR^{WT} and 32D-MPL-CALR^{del52} cells after overnight IL-3 withdrawal. Cells were fixed, permeabilized and stained for phospho-STAT3 (Tyr705). Nuclei were stained with DAPI. Top row shows the merged images with a detailed section. Bottom row shows the single channel images (dapi and pSTAT3-Alexa fluor™ 647) of the respective merged image. Scale bar showing 50 µm (main pictures) and 25 µm (detailed section).

Supplementary Figure S7: Impact of mTOR inhibition on TGF-β production:

(A, B) Western blot (A) and relative quantification (B) showing total- and phospho-mTOR with respective β-actin as loading control in 32D-MPL-CALR^{WT} or –MPL-CALR^{del52} cells, as indicated. Protein was isolated from four independent IL3 starvations. P values were calculated using Mann-Whitney test.

(C) Scatter plot showing fold change in MFI of L-TGF-β1 on 32D-MPL-CALR^{del52} after overnight treatment with indicated concentrations of mTOR inhibitor rapamycin. Pooled data from three individual experiments. P values were calculated using Kruskal-Wallis test.

(D, E) Representative Histogram (D) and scatter plot (E) showing fold change in MFI of L-TGF-β1 on 32D-MPL-CALR^{del52} cells after overnight treatment with indicated concentration of anti-TGF-β neutralizing antibody. Pooled data from three individual experiments. P values were calculated using Kruskal-Wallis test.

Supplementary Figure S8: anti-TGFβ antibody treatment reduces STAT3 and ERK signaling activity

(A, B) Western blot (A) and relative quantification (B) of phospho (Y705)-STAT3 / total-STAT3. Protein was isolated from 32D-MPL-CALR^{del52} cells that were treated with anti-TGFβ antibody or Isotype for 15 or 30 minutes. **(C, D)** Western blot (C) and relative quantification (D) of phospho-ERK / total-ERK. Protein was isolated from 32D-MPL-CALR^{del52} cells that were treated with anti-TGFβ antibody or Isotype for 15 or 30 minutes. Pooled data from three individual experiments. *P* values were calculated using ordinary one-way ANOVA.

Supplementary Figure S9: Impact of MPL-CALR$^{WT/ins5}$ on NF-κB signaling

(A) Heat map showing expression profile of MPL-CALR$^{WT/ins5}$ transduced 32D cells. Relative expression of NF-κB signaling pathway molecules are shown. Color codes represents the Z-score log2 intensity. RNA was harvested from four independent IL3-starved cell cultures. Differential gene expression analysis was performed with the linear model-based approach (limma R package).

(B-D) Western blot (B) and relative quantification of total NF-κB (C) and total IκBα (D). Protein was isolated from 32D-MPL-CALR$^{WT/del52}$ cells after overnight IL-3 starvation. Pooled data from three independent experiments. *P* values were calculated using Mann-Whitney test.

(E) Scatter plot showing fold change of TNFα expression in 32D-MPL-Calr^{del52} cells treated with indicated concentrations of NF-κB inhibitor (JSH-23).

Pooled data from three independent experiments. *P* values were calculated using Friedman test.

Supplementary Figure S10: **Impact of inhibitors downstream of ERK and/or PI3K on L-TGFβ1 expression**

(A-E) Scatter plot showing expression of L-TGFβ1 on 32D-MPL-Calr^{del52} cells after overnight treatment with selected transcription factor inhibitors downstream of ERK and/or PI3K. **(A)** AP-1 specific inhibitor (T-5224), **(B)** Elk-1 specific inhibitor (TAT-DEF-Elk), **(C)** CREB specific inhibitor (666-15), **(D)** FOXO1 specific inhibitor AS1842856 and **(E)** NF-κB specific inhibitor (JSH-23). Pooled data of three independent experiments. *P* values were calculated using Kruskal-Wallis test.

Supplementary Figure S11: MPL expression in human erythroid progenitor cells

(A) Histogram plots showing increased MPL surface expression in iPSC-derived CALR^{DEL52} mutated CD45$^+$CD71$^+$CD235a$^+$ erythroid progenitors in comparison to isogenic CALR^{WT} cells.

(B) Scatter plot showing relative MFI of MPL surface expression on CALR^{WT} or CALR^{DEL52} iPSC-derived CD45$^+$CD71$^+$CD235a$^+$ erythroid progenitor cells, fold change to MFI of unstained

control. $CALR^{DEL52}$ iPSC and CRISPR repaired $CALR^{WT}$ control were generated from one $CALR^{del52}$ MPN patient. Each dot represents an individual experiment. The *P* value was calculated using unpaired Student´s t test

Supplementary Figure S12: Progeny pathway annotation

Heat map showing fold change in wmean for a selected set of differentially regulated gene pathways. DecoupleR R package with the progeny annotation was applied to each set group (NBM, MF and sAML) from the public data set GSE214361 and each group was weighted against each other (4).

Supplementary Figure S13: Gating strategy for patient derived bone marrow samples

(A) Representative gating for viable, $CD45^{+}CD110^{+}$ cells in bone marrow derived monocytes of patient / healthy donor. **(B)** Representative gating for viable $CD3^{+}$ $CD4^{+}$ $CD25^{high}$ $FoxP3^{+}$ T_{reg} cells in bone marrow derived monocytes of patient / healthy donor.

Supplementary Figure S14: Graphical abstract

The simplified sketch shows the proposed mechanism of TGF-β mediated immune escape in $CALR^{del52}$ driven MPN. Secreted $CALR^{del52}$ binds to thrombopoietin receptor and induce intracellular downstream signaling via JAK/STAT, PI3K- and ERK-signaling. JAK/STAT signaling promotes cell survival and proliferation. PI3K and ERK activity promotes Sp1 activation which leads to transcription of TGF-β. Secreted and activated TGF-β has multiple effects on the BM microenvironment. It inhibits differentiation of naïve $CD8^{+}$ T cells towards effector T cells while promoting $CD4^{+}$ T cells to differentiate towards a T_{reg} phenotype (reviewed in (5)). TGF-β directly suppresses cytotoxic effector T cell function and thereby promotes immune escape. The figure was created using BioRender.com.

Supplementary Tables:

Supplementary Table S1 List of patient characteristics (Freiburg MPN cohort)

Internal NO.	Age at sampling [years]	Gender	Phenotype / Genotype	Disease Burden in BM	Depicted in Fig.
P13	55	Male	PV on spec, TET2 mutation,	1.3% $CD34^+$, $CD117^+$	7D, 8C
P14	69	Female	Post-PV-myelofibrosis, $JAK2^{V617F}$- and TET2 mutation	1.0% $CD34^+$, $CD117^+$	8A, 7D, 8C
P15	56	Female	MPN with thrombocytosis, $JAK2^{V617F}$- and MPL mutation	No blasts	8A, 7D, 8C
P16	63	Male	MPN/MDS, ASXL1, CBLB (2 Mutationen), CUX1, RUNX1 und SRSF2 mutations	1.0% $CD34^+$ bzw 3.0% $CD14^+$	8A, 7D, 8C
P17	31	Female	ET, JAK2 ^{V617F} mutation	2.5% $CD34^+$, $CD117^+$	8A, 7D, 8C
P18	66	Male	ET, JAK2 ^{V617F} mutation	1.0% $CD34^+$, $CD117^+$	8A, 7D, 8C
P19	85	Male	MPN with increased Megakaryopoiesis, $CALR^{del}$ and TET2 mutation	0,3% $CD34^+$ and 0,8% $CD117^+$	8A, 7D
P21	39	Female	MPN, $JAK2^{V617F}$ mutation	2% $CD117^+$ and partially $CD34^+$	8A
P22	78	Female	MPN with increase Erythro- and Megakaryopoiesis, ASXL1, $JAK2^{V617F}$ and TET2 mutation.	0.3% $CD34^+$, $CD117^+$	8A, 8C
P23	64	Male	sAML from MPN, $JAK2^{V617F}$ and RUNX1 mutation	10% $CD117^+$ and partially $CD34^+$, 2nd population 40% $CD14^+$	8A, 8C
P24	64	Male	MPN with Erythro- and Megakaryo- and Leukopoiesis, $JAK2^{V617F}$ mutation	2.5% $CD34^+$, $CD117^+$	8A, 8C
P40	38	Male	$Calr^{del52}$ and TET2 mutation	Mutation in 44% of BM cells	8A
P44	72	Male	ET with $Calr^{del52}$ and TET2 mutation	$Calr^{del52}$ 59% VAF	8A, 8C

Supplementary Table S2 Antibodies used for: Flow cytometry – Western blot – in-vivo

Flow cytometry antibodies						
**Target**	**Label**	**Reactivity**	**Isotype**	**Dilution**	**RRID or Cat. No**	**Vendor**
LAP(TGF-β1)	PE	Mouse	Mouse IgG1, κ	1:50	AB_10720866	BioLegend
CD45	BV785	Mouse	Rat IgG2b, κ	1:100	AB_2564590	BioLegend
Lineage cocktail	PB	Mouse	Rat IgG2b, Rat IgG2a	1:50	AB_11126168	BioLegend
CD34	Percp-Cy5.5	Mouse		1:50	AB_2260023	BioLegend
CD38	APC	Mouse	Rat IgG2a,k	1:50	AB_312933	BioLegend
CD90	PE-Cy7	Mouse	Rat IgG2b, k	1:50	AB_2201290	BioLegend
Perforin	APC	Mouse	Rat / IgG2a, kappa	1:100	AB_469514	invitrogen
Granzyme B	FITC	Mouse	Rat / IgG2a, kappa	1:100	AB_10732989	invitrogen
TNF-α	PE	Mouse	Rat IgG1, kappa	1:100	AB_466198	invitrogen
CD8	PB	Mouse	Rat IgG2a, k	1:100	AB_493425	BioLegend
CD4	BV510	Mouse	Rat IgG2b, κ	1:100	AB_2564587	BioLegend
Foxp3	APC	Mouse	Rat IgG2a kappa	1:100	AB_469457	eBioscience
CD25	PE	Mouse	Rat IgG1, λ	1:100	AB_312856	BioLegend
CD3	PB	Mouse	Rat IgG2b,k	1:100	AB_493644	BioLegend
CD8	APC-H7	Mouse	Rat IgG2a, κ	1:100	AB_1645237	BD Pharmingen
CD69	APC	Mouse	Armenian hamster / IgG	1:100	AB_1210795	eBioscience
CD3	PE-Cy7	Mouse	Rat IgG2b, k	1:100	AB_1732057	BioLegend
CD4	FITC	Mouse	Rat IgG2b, k	1:100	AB_312690	BioLegend
Foxp3	eF450	Mouse	Rat / IgG2a, kappa	1:50	AB_1518812	eBioscience
CD8	APC	Mouse	Rat IgG2a, k	1:100	AB_469336	eBioscience

LAP(TGF-β1)	PE	Human	Mouse IgG1, κ	1:25	AB_10645520	BioLegend
CD45	PB	Human	Mouse IgG1,k	1:100	AB_2174123	BioLegend
CD110	APC	Human	Mouse IgG2b, κ	1:50	AB_2750426	BioLegend
CD3	PB	Human	Mouse IgG1, κ	1:100	AB_2563422	BioLegend
CD4	BV510	Human	Mouse IgG1, k	1:100	AB_2566017	BioLegend
CD8	Alexa Fluor(R) 488	Human	Mouse IgG1, k	1:100	AB_756152	BioLegend
Foxp3	PE	Human	Rat IgG2a, kappa	1:100	AB_1518782	eBioscience
CD25	APC	Human	Mouse IgG1, κ	1:100	AB_314280	BioLegend
CD71	FITC	Human	Mouse BALB/c IgG2a, κ		AB_10896151	BD Pharmingen
CD235a	PE	Human	Mouse IgG2a		21812354	Immunotool
**Western blot and immune fluorescence antibodies**						
**Target**	**Molecular weight [kDa]**	**Reactivity**	**Isotype**	**Dilution / block buffer.**	**RRID or Cat. No.**	**Vendor**
Actin	45	Mouse	Rabbit IgG	1:2000, 5% BSA	AB_2223172	Cell Signaling
Actin 8H10D10	45	Mouse	Mouse IgG	1:2000, 5% BSA	AB_2242334	Cell Signaling
STAT3	79; 86	Mouse	Rabbit IgG	1:1000, 5% BSA	AB_331269	Cell Signaling
Phospho-STAT3 (Tyr705)	79; 86	Mouse	Rabbit IgG	1:1000, 5% BSA	AB_331586	Cell Signaling
ERK (p44/42)	42; 44	Mouse	Rabbit IgG	1:1000, 5% BSA	AB_330744	Cell Signaling
Phospho- ERK (Thr202/ Tyr204)	42; 44	Mouse	Rabbit IgG	1:1000, 5% BSA	AB_331646	Cell Signaling
mTOR (7C10)	289	Mouse	Rabbit IgG	1:1000, 5% BSA	AB_2105622	Cell Signaling
Phospho mTOR (Ser2448)	289	Mouse	Rabbit IgG, D9C2	1:1000, 5% milk	AB_10691552	Cell Signaling

Goat anti Mouse IgA Secondary Antibody, HRP	secondary	Anti-mouse IgG	Goat IgA – HRP linked	1:2000 – 1:4000, 5% BSA or milk	AB_138466	Thermo Fisher Scientific
Anti rabbit IgG, HRP linked Antibody	secondary	Anti rabbit IgG	Goat, HRP linked	1:2000 – 1:4000, 5% BSA or milk	AB_2099233	Cell Signaling
Goat-anti-rabbit IgG (H+L) AlexaFluorPlus 647	secondary	Anti-rabbit	AlexaFluor647	1:100, 5% BSA and % Goat Serum	AB_2633282	Invitrogen
**In vivo antibodies**						
**Target**	**Clone**	**Reactivity**	**Isotype**	**Concent.**	**RRID or Cat.No.**	**Vendor**
Bulk anti-TGF-B1,2,3 In Vivo	1D11.1	Human, Mouse, Bovine, Chicken	Mouse IgG1	10µg/g_{BW}	AB_2921436	Ichorbio
Mouse IgG1 Isotype Control	HKSP	-	Mouse IgG1	10µg/g_{BW}	AB_2921382	Ichorbio
Anti-mouse CD8 *in vivo*	YTS-169	Mouse	Rat IgG2b	12,5µg/g_{BW}	AB_2921445	Ichorbio
Anti-mouse CD4 *in vivo*	GK1.5	Mouse	Rat IgG2b	12,5µg/g_{BW}	AB_2921444	Ichorbio
Rat IgG2b Isotype Control *in vivo*	1-2	-	Rat IgG2b	12,5µg/g_{BW}	AB_2921378	Ichorbio

Supplementary Table S3: Primers used for RT-qPCR

Target gene	Direction	Sequence (5'→3')	GC [%]	Length	T_M
hu-TGF-β1	Fwd	ATTCCTGGCGATACCTCAGC	55	20	59,4
hu-TGF-β1	Rev	CGGTAGTGAACCCGTTGATG	55	20	59,4
hu-GAPDH	Fwd	GTCAGTGGTGGACCTGACCT	60	20	61,4
hu-GAPDH	Rev	TGAGCTTGACAAAGTGGTCG	50	20	57,3

Supplementary Table S4: Expression vectors

Name	Backbone	Insert (cDNA)	Selection marker	RRID/parentage
pMIG	pMSCV	empty	IRES-GFP	RRID:Addgene_9044
pMIG-$CALR^{WT}$	pMSCV	Human $CALR^{WT}$	IRES-GFP	Provided by Ann Mullally
pMIG-$CALR^{ins5}$	pMSCV	Human $CALR^{ins5}$	IRES-GFP	Provided by Ann Mullally

pMIG-CALR^{del52}	pMSCV	Human *CALR^{del52}*	IRES-GFP	Provided by Ann Mullally
pMSCV-MPL	pMSCV	Human *MPL*	PKG-hygromycin resistance gene	Provided by Ann Mullally
pMIG-JAK2^{WT}	pMSCV	Human *JAK2^{WT}*	IRES-GFP	Provided by Justus Duyster
pMIG-JAK2^{V617F}	pMSCV	Human *JAK2^{V617F}*	IRES-GFP	Provided by Justus Duyster
pMSCV-EpoR	pMSCV	Human *EPOR*	neomycin resistance gene	In-house
pMIG-STAT3^{WT}	pMX	Human *STAT3^{WT}*	IRES-GFP	In-house
pMIG-STAT3^{V640F}	pMX	Human *STAT3^{V640F}*	IRES-GFP	In-house
pGL4.73[hRluc/SV40]	pGL4	SV40 early enhancer, *rRluc*	renilla luciferase	Promega (E691A)
pGL3-TGFb1	pGL3	Human TGFb1 promoter	luciferase	RRID:Addgene_ 101762

References:

1. Stumpf PS, Du X, Imanishi H, Kunisaki Y, Semba Y, Noble T, *et al.* Transfer learning efficiently maps bone marrow cell types from mouse to human using single-cell RNA sequencing. Communications biology **2020**;3:736
2. Harris BD, Lee J, Gillis J. A meta-analytic single-cell atlas of mouse bone marrow hematopoietic development. BioRxiv **2021**:2021.08. 12.456098
3. Lee RD, Munro SA, Knutson TP, LaRue RS, Heltemes-Harris LM, Farrar MA. Single-cell analysis identifies dynamic gene expression networks that govern B cell development and transformation. Nature communications **2021**;12:6843
4. Kong T, Laranjeira AB, Yang K, Fisher DA, Yu L, Poittevin De La Frégonnière L, *et al.* DUSP6 mediates resistance to JAK2 inhibition and drives leukemic progression. Nature Cancer **2023**;4:108-27
5. Dahmani A, Delisle J-S. TGF-β in T cell biology: implications for cancer immunotherapy. Cancers **2018**;10:194
